# Supplementary material for: Same-day testing with initiation of antiretroviral therapy or tuberculosis treatment versus standard care for persons presenting with tuberculosis symptoms at HIV diagnosis: A randomized open-label trial from Haiti
Source: PLoS Med. 2023 Jun 9;20(6):e1004246. doi: 10.1371/journal.pmed.1004246 (PMC10292694; doi:10.1371/journal.pmed.1004246)
Supplement: S1 Study Protocol — (DOCX) [file pmed.1004246.s001.docx]

**A Trial of Same-Day Testing and Treatment to Improve Outcomes among Symptomatic Patients Newly Diagnosed with HIV**

**Protocol Chairs:**

Serena Koenig, MD

Jean William Pape, MD

Patrice Severe, MD

**Version Number:** 3, LOA #2

**Date:** December 18, 2017

Table of Contents

Table of Contents 2

1. Key Roles 4

2. List of Abbreviations 8

3. Protocol Summary 9

4. Introduction 12

4.1 Background Information 12

4.2 Rationale 14

4.3 Study Hypothesis 15

5. Objectives 15

5.1 Primary Objective 15

5.2 Secondary Objectives 15

6. Study Design 16

7. Study Population 17

7.1 Inclusion and Exclusion Criteria 17

7.1.1 Participant Inclusion Criteria 17

7.1.2 Participant Exclusion Criteria 17

7.2 Recruitment Processes 18

7.3 Participant Retention 18

8. Study Intervention 18

9. Study Procedure/Evaluations 19

9.1 Clinical Evaluations and Procedures 19

9.2 Laboratory Evaluations 23

9.2.1 Special Assays or Procedures 23

9.2.2 Specimen Preparation, Handling, and Shipping………………………………… 24

9.2.3 Biohazard Containment 25

9.2.4 Total Blood Volume 25

9.3 Schedule of Procedures/Evaluations 26

9.3.1 Screening 26

9.3.2 Enrollment 26

9.3.3 Randomization Procedures 27

9.3 4 Follow Up Visits 27

9.3.5 Reasons for Withdrawal 27

9.3.6 Handling of Withdrawals 28

9.3.7 Final Study Visit 28

10. Assessment of Safety 28

10.1 Safety Assessment Overview 28

10.2 Adverse Event Procedures and Reporting Requirements 29

11. Clinical Management 30

12. Statistical Analysis Plan 33

12.1 Overview and General Design Issues 33

12.2 Study Endpoints 33

12.2.1 Primary Endpoints 33

12.2.2 Secondary Endpoints 33

12.3 Study Objectives and Hypotheses 35

12.4 Sample Size Considerations 35

12.5 Enrollment and Randomization Procedures 36

12.6 Data and Safety Monitoring 37

12.7 Analysis Plan 37

13. Data Handling and Record Keeping 39

13.1 Data Management Responsibilities 39

13.2 Essential/Source Documents 39

13.3 Quality Control and Quality Assurance 40

14. Clinical Site Monitoring 40

15. Human Subjects Protection 40

15.1 Institutional Review Board/Ethics Committee 40

15.2 Vulnerable Populations 40

15.3 Informed Consent Process 41

15.4 Risks 42

15.5 Social Impact Events 42

15.6 Benefits 43

15.7 Compensation 43

15.8 Participant Privacy and Confidentiality 43

15.9 Study Discontinuation 44

16. Adminstrative Procedures 44

17 Literature References 45

# 1. Key Roles

**Protocol Chairs:**

Serena Koenig, MD

Jean William Pape, MD

Patrice Severe, MD

**2. LIST OF ABBREVIATIONS**

AFB acid fast bacilli

ALT alanine aminotransferase

ART antiretroviral therapy

AST aspartate aminotransferase

CBC complete blood count

CHW community health worker

EFV efavirenz

INH isoniazid

IRIS immune reconstitution inflammatory syndrome

LTFU lost to follow-up

OI opportunistic infection

PCP Pneumocystis jiroveci pneumonia

SMZ co-trimoxazole

TB tuberculosis

WHO World Health Organization

**3. PROTOCOL SUMMARY**

**Title:** A Trial of Same-Day Testing and Treatment to Improve Outcomes among Symptomatic Patients Newly Diagnosed with HIV

**Study Design:** Randomized, open-label study comparing standard vs. same-day treatment for patients with TB symptoms (cough, fever, night sweats, or weight loss) at HIV diagnosis.

**Study Duration:** 3 years

**Subject Participation Duration:** 48 weeks

**Sample Size:** 600 patients (300 per group)

**Site**: The Haitian Group for the Study of Kaposi's Sarcoma and Opportunistic Infections (GHESKIO), Port-au-Prince, Haiti

**Study Population:** The study population includes HIV-infected men and women ≥18 years of age who are ART-naïve, and who present with symptoms of TB (cough, fever, nights sweats, or weight loss) at HIV diagnosis.

**Randomization:** 1:1 to standard or same-day treatment

**Study Schema:**

Study Entry

Same-Day Treatment

Standard Treatment

**Study Intervention:**

Standard Care: On the day of enrollment (Day 0), participants in the standard group will receive evaluation by the study physician, with a blood draw for routine baseline ART tests, spot sputum for Xpert Ultra testing, and a chest x-ray. Participants with a clinical syndrome and chest x-ray that are highly suspicious for TB will start same-day TB treatment. Participants will also receive diagnostic testing and empiric treatment for other OIs. All participants in the standard group will return on Day 2 for CD4 count and Xpert Ultra results, and to provide a second (early morning) sputum sample. Liquid culture will be performed on both spot and early morning specimens. Those who are Xpert Ultra positive will receive same-day (Day 2) TB treatment, if not started on Day 0. Participants who are diagnosed with TB at the Day 0 or Day 2 visit will return at Week 2, for evaluation and ART initiation, and then for physician visits at Weeks 4, 6 and 8 (as is standard of care during the intensive phase of TB treatment at GHESKIO). Participants who are not diagnosed with TB will receive additional testing and treatment for other OIs as clinically indicated, and return on Day 7 for follow-up evaluation and ART initiation; they will have follow-up physician visits during Weeks 3 and 8, with subsequent visits outlined in the Table of Events (see Appendix 1).

Same-Day Treatment Group: On the day of enrollment (Day 0), participants in the same-day group will receive evaluation by the study physician, with blood draw for routine baseline tests, as for the standard group. Spot sputum for Xpert Ultra testing will be conducted with same-day results; specimens will also be sent for liquid culture. Same-day chest x-ray will also be conducted; those with negative Xpert Ultra but clinical symptoms and chest x-ray that are highly suspicious for TB will be empirically treated for TB. Participants will also receive additional diagnostic testing and treatment for other OIs as clinically indicated. Participants will be treated with either ART or TB medication (Day 0). Participants will return on Day 2 to drop off an early morning specimen at the laboratory for repeat sputum testing, and for a short study visit to receive CD4 count results. Participants who have not been diagnosed with TB will return at Weeks 2, 4, and 8 for a physician evaluation to assess clinical status and provide adherence counseling. Those who are diagnosed with TB will return at Week 2 for a follow-up visit, and to initiate ART; and then for physician visits at Weeks 4, 6, and 8 (as for the standard group), with subsequent visits outlined in the Table of Events (see Appendix 1).

**Primary Objective:**

- To compare the proportion of participants in the standard and same-day treatment groups who are alive and in care with a plasma HIV-1 RNA level <200 copies/ml at 48 weeks after enrollment

**Secondary Efficacy Objectives:**

- To compare mortality at 48 weeks after enrollment, and time to death, between standard and same-day treatment groups
- To compare the proportion of participants with HIV-1 RNA level <50 copies/ml and <1000 copies at 48 weeks after enrollment in each group
- To compare the proportion of participants in each group with 48-week adherence to ART ≥90%, as measured by medication possession ratio (pharmacy refill records)
- To document the proportion of participants in each group at the 2-week, 12-week, 24-week, and 48-week visit who report that the last missed dose of medication was at least 2 weeks ago, and to correlate with 24 and 48-week viral load results
- To compare the proportion of participants in each group who develop WHO criteria for ART failure, and the proportion of patients who start second-line ART
- To calculate the sensitivity, specificity, predictive values, and likelihood ratios of spot and early morning Xpert Ultra tests and chest x-ray as single and as combined tests, with liquid culture as gold standard
- To calculate the cost and cost-effectiveness of standard and same-day treatment from the health center perspective
- To calculate the median time spent in clinic during the first day and month of the study
- To compare the mean score on the Connectedness to Treatment Setting Scale, the State Hope Scale, and the COPE survey at 2 weeks, 12 weeks, 24 weeks, and 48 weeks after HIV testing, and to compare the mean score on the HRSA Patient Satisfaction Survey at 12, 24, and 48 weeks after HIV testing, between the two groups.
- To identify barriers and facilitators to treatment initiation, clinic attendance and adherence through in-depth interviews with patients in the standard care and same-day treatment groups.
- To measure the baseline prevalence of diarrhea (Day 0), and the incidence of diarrhea during the 48-week study period in both groups.
- Among participants in both groups who report diarrhea, to administer a GHESKIO questionnaire to determine the frequency of diarrhea and the impact on work and other activities.

**Safety Outcomes:**

- To compare the proportion of participants developing incident culture-positive TB after ART initiation (BACTEC MGIT 960, Becton Dickinson) in each group
- To compare the incidence of paradoxical or unmasking IRIS in each group
- To compare the proportion of participants who develop new Division of AIDS Grade 3 or Grade 4 signs, symptoms, or laboratory abnormalities that are at least a one-grade increase from baseline in each group.

# 4. INTRODUCTION

## 4.1 Background Information

Attrition rates are high prior to ART initiation.^1-20^ In a review of 28 African studies a median of 59% of patients completed CD4 testing (range 35-88%) and 68% (range 14-84%) of qualifying patients initiated ART.^2^ A meta-analysis of 29 African studies found that CD4 count was measured in 78% of patients and 63% initiated ART.^20^ Pre-ART attrition rates are 14 to 26% even in those starting ART within 2 to 4 weeks of HIV testing, and delays in ART initiation are associated with mortality, particularly among those with advanced disease.^1-20^ Predictors of pre-ART attrition include male sex, younger age, low socio-economic status, poor trust in services, long waiting times, inability to meet clinic requirements (medicine companion, disclosure), stigma, and distance from treatment sites.^1,3,9-14,21-23^ Retention in care and adherence after ART initiation are also sub-optimal.^13,15,24-30^ A meta-analysis of 39 African cohorts found attrition rates of 20% at 12 months.^28^ Predictors of ART attrition include male gender, low socio-economic status, and advanced disease.^29,31,32^ Obstacles to retention include transportation costs and distance to clinic, competing demands for time, long clinic waiting times and inconvenient hours, stigma and fear of disclosure.^21,32,33^

*Three studies have evaluated accelerated ART initiation in resource-poor settings, and have found it to be both feasible and effective.^34-36^* One is the same-day ART study conducted at GHESKIO, which included patients with WHO Stage 1 or 2 disease and CD4 count ≤500 cells/mm^3^.^34^ All participants received same-day HIV and CD4 count testing, TB screening, chest x-ray, and counseling on the day of presentation. The standard group received follow-up visits with physician and social worker on Days 7, 14, and 21, and started ART on Day 21; the same-day ART group started ART on the day of presentation. The only difference between groups was the timing of ART initiation and visit schedule for the first month of the study. The primary outcome was the proportion of participants who were retained in care at 48 weeks after HIV testing with a HIV-1 RNA <50 copies/ml; secondary outcomes included proportion who started ART, mortality, and HIV-1 RNA <200 copies/ml. From August 5, 2013 to February 28, 2015, 564 patients were enrolled (285 in the standard and 279 in the same-day group). The median age was 37 years (IQR: 30-45 years), 275 (49%) were women, and the median CD4 count was 242 cells/mm^3^ (IQR: 141, 333). Baseline characteristics were similar between the two groups, except that the standard group had higher body mass index compared to the same-day group (21.6 versus 20.8; p=0.020). Of the 285 participants in the standard group, 201 (71%) were retained in care, 19 (7%) died, 43 (15%) were LTFU, and 22 (8%) were late returners. Among the 201 participants retained in the standard group, 120 (60% of retained and 42% overall) had HIV-1 RNA <50 copies/ml. Of the 279 participants in the same-day ART group, 224 (80%) were retained in care, 8 (3%) died, 34 (12%) were LTFU, and 13 (5%) were late returners. Among the 224 participants retained in the same-day ART group, 151 (67% of retained and 54% overall) had HIV-1 RNA <50 copies/ml. The adjusted OR of being retained in care at 48 weeks and achieving HIV-1 RNA <50 copies/ml was 1.76 (95% CI: 1.24, 2.49; p=0.002) for the same-day ART compared to the standard group.

The second study is the RapIT randomized trial from South Africa, which included patients with CD4 count ≤350 cells/mm^3^.^35^ They compared standard ART initiation, which generally required 6 pre-ART visits, with rapid ART initiation, which included point-of-care CD4 count, TB symptom screen, and counseling on the day of enrollment. They found that 136 of 190 (72%) participants in the standard group and 182 of 187 (97%) in the rapid group initiated ART within the subsequent 90 days, including 73% on the same day as study enrollment (crude RR 1.36; 95% CI: 1.24-1.49). Among all participants enrolled, 121 (64%) in the standard and 151 (81%) in the rapid group were retained in care at 10 months; 96 (51%) in the standard and 119 (64%) in the rapid group were retained in care at 10 months with viral load ≤400 copies/ml (crude RR: 1.26; 95% CI: 1.05-1.50). The third study is START, which was conducted in 20 clinics in Uganda with a step-wedge design, with targeted knowledge transfer, installation of point-of-care CD4 count machines, and feedback to clinics regarding timing of ART.^36^ Among 12,024 eligible patients, 7277 were managed during the control period, and 4747 were managed during the intervention period. The proportion of participants starting ART within the subsequent 90 days was 70% in the standard and 90% in the intervention group (RR 1.27; 95% CI: 1.25-1.30); 71% of intervention group patients received same-day ART. Among a random sample of participants, there was no difference if 12-month viral load tests not done were counted as failures (58% vs. 66%; RR 1.14; 95% CI: 0.93-1.38), but among those with viral load completed, 71% in the standard and 86% in the intervention group had <200 copies/ml (RR 1.22; 95% CI: 1.05-1.41).

Xpert has revolutionized the diagnosis of TB, with more than 16 million tests conducted in 122 countries since 2011. Xpert testing has an overall sensitivity of about 75-80% in HIV-infected patients; in smear-negative patients, the sensitivity of Xpert is about 60-80%.^37-39^ The sensitivity is higher with two samples, compared with one sample, and with morning specimens.^40-43^ The sensitivity of Xpert is also greater in patients with a poorer prognosis.^44^ In a study from South Africa, patients who were Xpert-negative, culture-positive had higher CD4 counts, hemoglobin and body mass index, less extensive radiographic abnormalities, and lower mortality, compared with Xpert-positive, culture-positive patients, even though they were more likely to start TB medication after ART initiation, when cultures turned positive.^43^ However, the impact of Xpert has been limited by weak health systems,^45^ as recently defined by Albert et al: 1) inadequate screening and referral for testing; 2) delays in provision of test results to providers; 3) inefficient processes to recall patients and pre-treatment LTFU; and 4) need for referral and/or confirmatory testing in some settings.^46^ In many countries, data on pre-treatment LTFU and time to treatment initiation are not collected, and in many settings, the proportion of patients who are diagnosed and then LTFU prior to treatment is substantial, even with Xpert testing. Up to 30% of patients with TB are lost prior to starting treatment, even when TB treatment is initiated within a week after presentation, unless same-day results are provided.^38,45,47^ Ultra is a soon-to-be-available next-generation Xpert test. It includes a new sample processing cartridge that doubled the amount of purified DNA delivered to the PCR reaction.^48^ Four newly designed probes that detected mutations in the *rpoB* gene replaced the 4 Xpert real-time probes, real-time *Mtb* detecting probes targeting IS6110 and IS1081 were added, and cartridge fluidics and PCR cycling were optimized. With sputum samples spiked with *Mtb* H37Rv, Ultra had a level of detection of 5 CFU/ml, compared to 50 CFU/ml for Xpert (p=0.001). Ultra will be commercially available in March 2017, and it is expected that it will replace Xpert within the next 1 to 3 years.

One potential concern with accelerated ART initiation in symptomatic patients is the development of IRIS, a clinical syndrome characterized by an excessive inflammatory response after the initiation of ART. Paradoxical IRIS is due to worsening of appropriately treated disease after the initiation of ART. Unmasking IRIS occurs when the disease is missed at ART initiation due to subclinical or atypical presentation, and becomes clinically evident, with a profuse immunologic response, after the immune system is able to mount a specific defense.^49^ Pulmonary and extra-pulmonary TB are the leading causes of IRIS in resource-poor settings.^50^ TB is frequently unrecognized at ART initiation, leading to high rates of TB diagnosis during the first 3 months after ART, particularly among patients with low CD4 counts, who are more likely to present with subclinical disease or atypical symptoms.^49,51,52^ Studies from South Africa and Zambia found that about 20% of patients undergoing ART staging had undiagnosed, culture-positive TB.^53,54^ Active disease then becomes clinically evident only after the immune system is able to mount a specific defense.^55^ Missed TB at ART initiation may result in increased mortality.^56^ Other causes of IRIS include cytomegalovirus (CMV), hepatitis B and C, Cryptococcus, and Kaposi’s sarcoma.

## Rationale

UNAIDS has set global targets of diagnosing 90% of people living with HIV, initiating antiretroviral therapy (ART) for 90% of those found to be infected, and achieving virologic suppression in 90% of those on ART by the year 2020 to curb the AIDS epidemic.^57^ To achieve this goal, patients must be promptly linked to HIV services, initiated on ART, and retained in lifelong care. Attrition rates are particularly high during the period from HIV testing to ART initiation, as patients are lost to care during the multiple visits required to initiate ART.^1-20^ To improve retention, same-day HIV testing and ART initiation is now being scaled up for patients with early clinical disease in many settings, after superior outcomes have been demonstrated with this strategy, compared with standard care.^34,35,58^ In resource-poor settings with fragile delivery systems, the provision of immediate support by care providers at the time of HIV diagnosis can have both structural and individual impact. In addition to making treatment initiation logistically easier for patients, we believe that same-day counseling and ART initiation increase the sense of hope, optimism, and overall connectedness to the health care system for patients, which has been shown to be important for retention.^59-62^ However, about one-third of patients present for HIV testing with cough, fever, night sweats, or weight loss.^63^ Due to concerns about starting ART in the presence of undiagnosed TB, and suboptimal sensitivity of Xpert for the diagnosis of smear-negative TB, same-day treatment has not been systematically evaluated in this population.However, a next-generation Xpert Ultra test cartridge has been developed, which will be commercially available in March 2017 for use in Xpert machines.^48^ Ultra has a limit of detection that is 10-fold lower than the Xpert, with a sensitivity of >95% in clinical trials. If these results are replicated in real-world conditions, it will make same-day testing and treatment in symptomatic patients feasible. However, before implementing this strategy, further study is required to demonstrate that retention in care and viral suppression rates are higher with same-day treatment, compared with a one to two-week period for additional testing to rule out TB and other opportunistic infections OIs prior to ART initiation. It will also be important to evaluate rates of missed TB and immune reconstitution inflammatory syndrome IRIS with this strategy of same-day TB testing and concurrent initiation of ART and management of non-TB causes of systemic symptoms (such as pneumonia and other bacterial infections). Furthermore, an evaluation of the cost-effectiveness of same-day testing and treatment is necessary, because same-day services require extra clinical and laboratory resources.

## Study Hypotheses

We hypothesize that same-day treatment with either ART or TB medication will result in a higher rate of retention with viral suppression and lower rate of mortality, compared with standard care, and that it will be cost-effective for use in Haiti and other low-income countries.

**5. OBJECTIVES**

**5.1 Primary Objective:**

- To compare the proportion of participants in the standard and same-day treatment groups who are alive and in care with a plasma HIV-1 RNA level <200 copies/ml at 48 weeks after enrollment

**5.2 Secondary Efficacy Objectives:**

- To compare mortality at 48 weeks after enrollment, and time to death, between standard and same-day treatment group
- To compare the proportion of participants with HIV-1 RNA level <50 copies/ml and <1000 copies at 48 weeks after enrollment in each group
- To compare the proportion of participants in each group with 48-week adherence to ART ≥90%, as measured by medication possession ratio (pharmacy refill records)
- To document the proportion of participants in each group at the 2-week, 12-week, 24-week, and 48-week visit who report that the last missed dose of medication was at least 2 weeks ago, and to correlate with 24 and 48-week viral load results.
- To compare the proportion of participants in each group who develop WHO criteria for ART failure, and the proportion of patients who start second-line ART
- To calculate the sensitivity, specificity, predictive values, and likelihood ratios of spot and early morning Xpert Ultra tests and chest x-ray, as single and as combined tests, with liquid culture as gold standard
- To calculate the cost and cost-effectiveness of standard and same-day treatment from the health center perspective
- To calculate the median time spent in clinic during the first day and month of the study
- To compare the mean score on the Connectedness to Treatment Setting Scale, the State Hope Scale, and the COPE survey at 2 weeks, 12 weeks, 24 weeks, and 48 weeks, and to compare the mean score on the HRSA Patient Satisfaction Survey at 12, 24, and 48 weeks after HIV testing in each group.
- To identify barriers and facilitators to treatment initiation, clinic attendance and adherence through in-depth interviews with patients in the standard care and same-day treatment groups.
- To measure the baseline prevalence of diarrhea (Day 0), and the incidence of diarrhea during the 48-week study period in both groups.
- Among participants in both groups who report diarrhea, to administer a GHESKIO questionnaire to determine the frequency of diarrhea and the impact on work and other activities.

**5.3 Secondary Safety Objectives:**

- To compare the proportion of participants developing culture-positive TB which is diagnosed after ART initiation (BACTEC MGIT 960, Becton Dickinson) in each group.
- To compare the incidence of paradoxical or unmasking IRIS in each group.
- To compare the proportion of participants who develop new Division of AIDS Grade 3 or Grade 4 signs, symptoms, or laboratory abnormalities that are at least a one-grade increase from baseline in each group.

**6. STUDY DESIGN**

Participants will be screened, enrolled, and randomized within 24 hours after receiving their HIV diagnosis. HIV testing will be conducted according to Ministry of Health guidelines and GHESKIO standard care. After the first HIV test is positive, a second confirmatory test will be conducted on a specimen from a second blood draw, to minimize the risk of false positive HIV test results. Participants will be eligible if they are enrolled in the study within 24 hours after receiving their HIV diagnosis.

Participants will be randomized to the standard or same-day groups in a 1:1 ratio. A data manager in the GHESKIO IT unit who is not involved in the study will conduct randomization by using a computer-number generator, using permuted blocks with variable block sizes from 2 to 6, with blinding to allocation. Block sizes will be selected in random order, with 50 blocks of size 6, 50 blocks of size 4, and 50 blocks of size 2.

The standard group will receive standard of care, which includes same-day chest x-ray, and 48-hour turn-around-time for Xpert Ultra testing; those with negative TB test results will receive sequential visits for the diagnosis and treatment of other OIs prior to ART initiation. The same-day group will receive same-day Xpert Ultra and chest x-ray, with a same-day decision to start either TB medication or ART. Participants with negative Xpert Ultra will have testing and treatment for other concurrent illnesses as clinically indicated (for example, a patient with diarrhea will have stool tests sent, and be treated empirically for diarrhea).

Participants in both groups will receive liquid culture and Xpert Ultra testing on both spot and early morning specimens. Xpert Ultra is a newly available test cartridge that is used with standard Xpert machines. It will be commercially available in March 2017. The Ultra cartridge has a limit of detection that is 10-fold lower than standard Xpert cartridges. A patient will be diagnosed with TB if either the Xpert Ultra or liquid culture (spot or early morning specimen) is positive. Furthermore, if a patient a clinical presentation and chest x-ray that is suspicious for TB, they will be treated empirically for TB, regardless of sputum testing results.

No participant will be enrolled until Institutional Review Boards at all participating institutions have approved it. Those involved will follow the Good Clinical Practice and Good Laboratory Management guidelines.

**7. STUDY POPULATION**

## 7.1 Inclusion and Exclusion Criteria

**7.1.1 Participant Inclusion Criteria**

- Men and women of ≥18 years of age
- Presence of cough, fever, night sweats, or weight loss, confirmed by participant and study physician
- Ability and willingness to give written informed consent
- Documentation of positive HIV status (test conducted at GHESKIO)
- Acceptance of HIV diagnosis, defined as affirmative responses to two questions: “I believe that HIV and AIDS exist” and “I believe that the results of my HIV test show that I am infected”.

**7.1.2 Participant Exclusion Criteria**

- Any use of ART in the past
- Treatment for TB in the year prior to screening visit
- Pregnancy or breastfeeding at the screening visit
- Active drug, alcohol use, or mental condition that would interfere with the ability to adhere to study requirements, in the opinion of the study physician
- Score of <3 for any of the 7 questions on the ART readiness survey
- Planning to transfer care to another clinic during the study period
- Symptoms consistent with WHO stage 4 neurologic disease (cryptococcal meningitis, TB meningitis; central nervous system toxoplasmosis; HIV encephalopathy; progressive multifocal leukoencephalopathy)
- Severe illness, classified as one of the WHO “danger signs” of temperature >39 degrees Celsius, pulse >120 beats/minutes, respiratory rate >30, or inability to walk unaided.

## Recruitment Process

All patients presenting to GHESKIO for HIV testing are screened with a symptom checklist at the time of HIV testing. Patients who test HIV-positive and report cough, fever, night sweats, or weight loss on the symptom checklist will be approached by the social worker and told about this study.

- 1. **Participant Retention**

Though this study will be conducted in the GHESKIO Clinical Trials Unit, participants will receive care that is similar to that provided to non-study patients, as our goal is to implement and evaluate a model of same-day treatment that could be scaled up in Haiti and other resource-poor settings. At each visit, participants will receive 100 Haitian gourdes ($US 1.70) for a transportation subsidy. If a patient fails to attend a scheduled visit, they will be phoned by a community health worker, as is standard of care at GHESKIO. If they cannot be reached by phone, a home visit will be attempted. At every visit, the study team will confirm address and phone number of participant and contacts, as is standard of care at GHESKIO.

1. **STUDY INTERVENTIONS**

This study compares a strategy of same-day treatment (ART or TB medications) with standard care among patients who present with TB symptoms (cough, fever, night sweats, or weight loss) at HIV testing. All diagnostic tests will be commercially available for TB testing at the time the study begins enrolling participants. All participants will receive standard medications, including TB medications and ART, as for non-study patients at GHESKIO. The study intervention is the use of these diagnostic tests to rule-in or rule-out TB on the day of HIV diagnosis, and then to initiate appropriate treatment (TB medication for those with TB, ART for those without TB). Participants who are diagnosed with TB will receive TB treatment, with ART deferred for two weeks, according to WHO guidelines and GHESKIO standard of care.

**9. STUDY PROCEDURES/EVALUATIONS**

**9.1 Clinical Evaluations and Procedures**

Standard Group – First Eight Weeks of Care: On the day of enrollment (Day 0), participants in the standard group will receive evaluation by the study physician, with blood draw for routine baseline ART tests (creatinine, aspartate transaminase [AST], alanine transaminase [ALT], complete blood count, and CD4 count) spot sputum for Xpert Ultra testing (Figure 1), and chest x-ray. Results of the chest x-ray will be available on Day 0; those with a clinical syndrome and chest x-ray that are highly suspicious for TB will receive same-day TB treatment. They will also receive diagnostic testing and treatment for other OIs, as clinically indicated. They will return on Day 2 for CD4 count and sputum test results, and to provide a second (early morning) sputum sample. Liquid culture will be performed on both spot and early morning specimens. Those who are Xpert Ultra positive will receive same-day (Day 2) TB treatment; all patients who are diagnosed with TB will also receive smear microscopy. In the standard group, participants will be diagnosed with TB through one of the following strategies, and TB treatment will be initiated on the day of TB diagnosis:

- Day 0: Clinical syndrome and chest x-ray highly suspicious for TB;
- Day 2: Positive Xpert Ultra from Day 0 (spot) sputum;
- Day 4: Positive Xpert Ultra from Day 2 (early-morning) sputum (no visit is scheduled, but patient will be phoned to return for treatment if Day 2 Xpert Ultra is positive);
- If TB has not been diagnosed, and at least one of the liquid culture results turns positive, participant will be contacted to return for TB treatment. If culture is negative, TB treatment will be completed if started.

Participants who are diagnosed with TB will initiate ART after a delay of 2 weeks, which is in accordance with WHO guidelines and GHESKIO standard of care.^64^ They will also have follow-up visits at Weeks 4, 6, and 8 (intensive phase of TB treatment), as is standard of care for patients with HIV-TB co-infection at GHESKIO. Participants who are not diagnosed with TB (an anticipated 75% of participants) will start ART on Day 7, after evaluation for TB and other OIs. They will receive a counseling session with a social worker prior to ART initiation. After initiating ART on Day 7, participants will return for physician visits during Weeks 3 and 8.

Same-Day Treatment Group: On the day of enrollment (Day 0), participants in the same-day group will receive evaluation by the study physician, with blood draw for routine baseline tests, as for the standard group. Spot sputum for Xpert Ultra testing and chest x-ray will be conducted with same-day results. Those with positive Xpert Ultra tests will receive TB treatment; those with negative Xpert Ultra but clinical symptoms and chest x-ray that are highly suspicious for TB (such as cavitary disease or hilar lymphadenopathy) will be empirically treated for TB. TB treatment will be provided on the day of diagnosis, and sputum will be sent for smear microscopy. All participants in the same-day group will return on Day 2 to drop an early morning sputum specimen in the lab for repeat sputum testing, and for a short study visit to receive CD4 count results. Those who had negative Xpert Ultra on the spot specimen (Day 0), but positive results on early morning specimen (Day 2) will be phoned to return to care immediately; ART will be stopped and TB medication will be started. Liquid culture will be performed on both spot and early morning specimens. Same-day treatment group participants who are diagnosed with TB will return at Week 2 (to start ART), and at Weeks 4, 6, and 8 (as is standard of care during the intensive phase of TB treatment).

Participants who are not diagnosed with TB will start ART on Day 0, after receiving adherence counseling. Participants will also receive additional testing and treatment for other OIs as clinically indicated. They will receive creatinine testing but results will not be available prior to ART initiation; those with CrCl <50 mL/minute will be phoned to return immediately for adjustment of ART (estimated 4% of patients). Follow-up visits will be scheduled during Weeks 2, 4, and 8.

Care for Both Groups: Both groups will also receive adherence counseling, which has been developed over more than a decade of providing ART at GHESKIO, and is based on a team approach involving the physician, nurse, pharmacist and social worker. This collaborative strategy was adapted from evidence-based approaches including cognitive behavioral therapy and motivational interviewing. The social worker will provide adherence counseling for about 30 minutes on the day of treatment initiation in both groups. This session will follow a standardized protocol that has been used in prior NIH-funded studies at GHESKIO, and it will be audiotaped for quality control. TB treatment will be provided according to WHO guidelines (rifampin, isoniazid, ethambutol, and pyrazinamide for 8 weeks, followed by rifampin and isoniazid for 16 weeks); pyridoxine will be provided throughout TB treatment. The ART regimen provided to both groups will be the same as for non-study patients; first-line therapy is efavirenz, tenofovir, and lamivudine or dolutegravir, tenofovir, and lamivudine. All participants will receive co-trimoxazole prophylaxis, which is recommended for all HIV-infected patients in Haitian guidelines, and those without active TB will receive isoniazid prophylaxis. After the first 8 weeks of the study, both groups will receive identical care, with visits at 8 and 12 weeks. Participants who are clinically stable and adherent at 12 weeks will receive a follow-up visit at 24 weeks; those who are clinically unstable or poorly adherent will continue monthly visits. At the 24-week visit, viral load testing will be conducted. Those with 24-week viral load ≤200 copies will continue quarterly visits. Those with >200 copies/ml will receive additional counseling, followed by repeat viral load at 36 weeks. Those with ≥1000 copies/ml on repeat testing after an adherence intervention will be changed to second-line ART, according to WHO guidelines.^64^ Throughout the study period, community health workers will make monthly phone calls for all participants to monitor for symptoms and adherence challenges. Both groups will receive a 100 gourdes ($1.70) transportation subsidy at each visit, and a phone call by a CHW if they miss a visit. A CHW will attempt a home visit for those who cannot be reached by phone.

Patients who transfer care to outside clinics will remain in the study, as long as they continue to provide informed consent. The study physician will contact the provider at the outside clinic to inform them that the participant is in a GHESKIO study, and a community health worker will phone the patient every month to check in. Participants will be provided with transportation fees to return to GHESKIO at Weeks 24 and 48 for study visits and viral load testing.

As a separate objective, we will measure the baseline prevalence of diarrhea in both groups on Day 0, and the incidence of diarrhea throughout the 48-week study period. At each visit, the clinician will query each participant about diarrhea; if the patient reports diarrhea, the clinician will administer a GHESKIO diarrhea questionnaire describing the frequency of diarrhea and impact on work and other activities.

**Figure 1: Study Interventions for the Standard and Same-Day Treatment Groups**

**
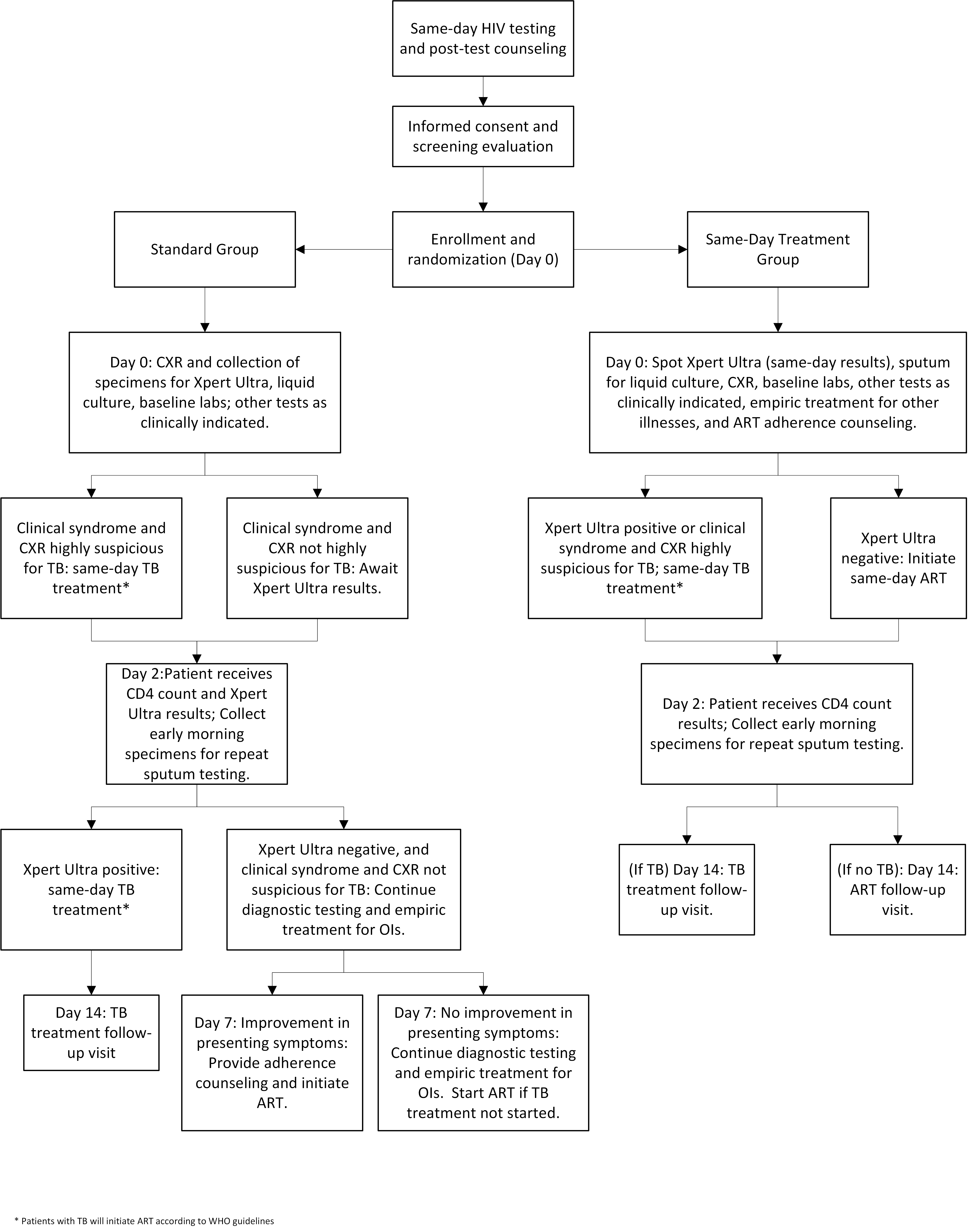
**

**9.2 Laboratory Evaluations**

**9.2.1 Special Assays or Procedures**

Researchers in a consortium of universities in the United States have developed an assay for rapid, specific, and high-sensitivity quantification of active TB disease by combining antibody-labeled, energy-focusing nanodisks with high-throughput mass spectrometry to enhance the detection of TB-specific peptides in digested samples.^65^ We propose to send these investigators a urine and serum specimen from consenting participants in our study population for further refinements and validation of their study technique. This will involve sending a urine and serum specimen for each consenting participant to Arizona State University.

Five urine metabolites diagnostic for active pulmonary TB were discovered in adult Haitian TB patients using mass spectrometry technology and validated in a second cohort from Vietnam. A combination of these five urine metabolites distinguished adults with active pulmonary TB from adults with other respiratory tract infections with a sensitivity of 92% and specificity of 92%. The biomarkers performed equally well in a pilot study of 15 HIV-infected adults with pulmonary TB compared to 15 HIV-infected adults without TB. Three of the urine metabolites are products of the host innate immune system, and two are breakdown products of the cell wall of *M. tuberculosis*. We propose to study these five urine metabolites. This will involve sending urine specimens to Weill Cornell Medical College.

A first episode of active TB is the most significant risk factor for a second episode of TB. At GHESKIO, HIV-infected people who have had TB before are twice as likely to be diagnosed with a TB recurrence than HIV-infected people who have never had TB. To define host susceptibility factors for TB recurrence, we propose to study levels of inflammation in people with HIV and TB to determine who is at highest risk for future TB recurrence by measuring immune transcript levels in the blood using transcriptomics. This will involve sending blood specimens to Weill Cornell Medical College.

*Note that none of these test results will be used for clinical purposes.*

Day 0:

- 1 mL of whole blood will be collected in a red top tube without additives, handled as described below (section 9.2.2) and shipped to Arizona State University.
- A C-reactive protein test will be conducted using whole blood from a finger stick, with the Alere Afinion rapid test system.
- 3 mL of whole blood will be collected in a blue top tube (Tempus), handled as described below, and shipped to Weill Cornell Medical College.
- Urine will be obtained via clean catch, and it will be stored at -20 degrees Celsius at GHESKIO, and shipped to Weill Cornell Medical College and Arizona State University (see section 9.2.2).

14 Days After ART Initiation: A C-reactive protein test will be conducted using whole blood from a finger stick, with the Alere Afinion rapid test system. From the same fingerstick, blood will be collected onto a Whatman Protein Saver 903 dried blood spot card for future testing related to HIV.

End of TB Treatment (if participant treated for TB): A C-reactive protein test will be conducted using whole blood from a finger stick, with the Alere Afinion rapid test system. From the same fingerstick, blood will be collected onto a Whatman Protein Saver 903 dried blood spot card for future testing related to HIV. 3 mL of whole blood will be collected in a Tempus tube.

**9.2.2 Specimen Preparation, Handling, and Shipment**

*Tests for Clinical Use*

All specimens will be handled following GHESKIO’s standard operating procedures. All testing for the clinical management of patients will be conducted locally, at GHESKIO. This includes baseline ART labs (creatinine, ALT, AST, complete blood count, and CD4 count) as well as HIV-1 viral load testing, which will be conducted at 24 and 48 weeks after HIV testing. Though a 24 and 48 week (6 and 12 month) viral load after ART initiation is recommended in Haitian national guidelines, financial resources are not always available to conduct these tests.

For the diagnosis of TB, spot and early morning sputum will be obtained according to GHESKIO protocol:

- Spot sputum: Patient is instructed how to cough deeply, and expectorate sample into a sterile sputum container. Sputum is kept in a cool box at 2-8° Celsius until it is transported to the laboratory. Once in the laboratory, sputum is prepped for studies.
- Morning Sputum: Patients are instructed to cough deeply upon first awakening and expectorate into a sterile container provided to patients the day prior. Patients are then instructed to return to clinic as soon as possible to hand in the sample. Sputum is kept in a cool box at 2-8° Celsius until it is transported to the laboratory.

*Research Tests (These Test Results will not be Used for the Clinical Management of Patients)*

- Blood: All samples will be obtained by the research nurse or phlebotomist. On Day 0, 1 mL specimen of blood will be collected in a red top tube without additives.  The blood sample will be centrifuged at the end of the clotting time (30-60 minutes) in a horizontal rotor (swing-out head) for 20 minutes at 1100-1300 g at room temperature. If the blood cannot be centrifuged immediately after the clotting time (30 to 60 minutes at room temperature), the tubes will be refrigerated (4ºC) for no longer than 4 hours.  A pipette will be used to transfer the serum, with an aliquot volume of 100 µl to 250 µl. This process will be completed within 1 hour of centrifugation. All aliquots will be placed upright in a specimen box or rack in an -80ºC or colder freezer. All specimens will remain at -80ºC or colder prior to shipping. These will be shipped to Arizona State University on dry ice.
- On Day 0, 3 mL of blood will be collected in a blue top Tempus tube. The blood sample will be shaken vigorously by hand or with a vortex mixer for 15 seconds immediately after phlebotomy. The tube will be frozen at -20C and shipped to Weill Cornell Medical College on dry ice. People with TB will also have 3 mL of whole blood collected in a Tempus tube after completing anti-TB treatment.
- Rapid C-reactive protein test with finger stick specimen: On Day 0, 14 days after ART initiation, and at completion of TB treatment (if participant treated for TB), the laboratory technician will conduct the C-reactive protein test with the use of the Alere Afinion test system, with a finger stick specimen (whole blood), according to manufacturers specifications (<http://www.alere.com/en/home/product-details/afinion-crp.html)>. From the same fingerstick, blood will be collected onto a Whatman Protein Saver 903 dried blood spot card for future testing related to HIV. 70 microliters of blood are applied to each of 5 circles on the card. They are allowed to air-dry and then added a sealed plastic bag with dessicant for long-term storage.
- Urine: On Day 0, urine will be collected via clean catch into a sterile urine cup. It will be transported at room temperature to the GHESKIO lab. It will be divided into two containers of 10 mL volume each, frozen within 3 hours of collection, and stored on dry ice. One specimen will be sent to Weill Cornell Medical College and one specimen will be sent to Arizona State University for analysis.

**9.2.3 Biohazard Containment**

Transmission of HIV and other blood borne pathogens can occur through contact with contaminated needles, blood, and blood products. Respiratory pathogens such a TB are transmitted by inhalation of droplet nuclei. Appropriate blood, secretion, and respiratory precautions will be employed by all personnel in the collection of clinical samples and the shipping and handling of all clinical samples and isolates for this study, as currently recommended by GHESKIO standard operating procedures, the NIH, the Centers for Disease Control and Prevention, and the World Health Organization.

**9.2.4 Total Blood Volume**

All blood tests conducted in this study are for standard clinical care, except extra tubes of blood will be drawn at baseline (total 4 mL) for all participants, and at 6 months (total 3 mL) for participants who complete TB treatment. A finger stick will be done on Day 0 and 14 days after ART initiation, and at completion of TB treatment, to conduct a C-reactive protein test and collect a dried blood spot card, as described above. Tests for standard clinical care include baseline ART labs (creatinine, ALT, AST, complete blood count, and CD4 count) as well as HIV-1 viral load testing, which will be conducted at 24 and 48 weeks after HIV testing. Though a 24 and 48 week (6 and 12 month) viral load after ART initiation is recommended in Haitian national guidelines, financial resources are not always available to conduct these tests. No safety labs are required for the purpose of the study itself – only for routine clinical care.

**9.3 Schedule of Procedures/Evaluations: Timing and Definitions**

**9.3.1 Screening**

Once identified as possible participants, social workers will give patients information regarding the study. Patients will have the opportunity to ask questions of the social worker. Social workers will go over the Informed Consent Form with patients in the patient’s native language (Creole). If after this discussion, patients are willing to proceed, they will complete an Assessment of Understanding quiz. Patients must receive at least 90% on the quiz to continue, as for all prospective studies that are conducted at GHESKIO. Once patient understanding of the study has been verified, patients will sign the Informed Consent Form.

Once the informed consent process is complete, patients will be given a unique Patient Identification Number. Patients will be screened for inclusion. Inclusion and exclusion criteria will be documented in a Screening CRF. Screening will include:

- Demographic questionnaire
- Medical and psychiatric history
- Assessment of alcohol and drug use
- History of any ART medication use in past, or TB within the last year
- Vital signs by study nurse
- Physical examination by study physician
- Confirmation of same-day positive HIV test result at GHESKIO
- Pregnancy testing for women <50 years of age

**9.3.2 Enrollment**

All inclusion and exclusion criteria will be verified by the research nurse and study physicians. If a patient meets all criteria, he/she will then be officially enrolled in the study. If a patient does not meet criteria, he/she will receive HIV and/or TB care through GHESKIO clinic free of charge per routine protocol.

### 9.3.3 Randomization Procedures

Participants will be randomized in a 1:1 ratio using a computer-generated random-number list in the Data Management Unit, as has been done in prior GHESKIO studies. A data manager in the GHESKIO IT unit who is not involved in the study will conduct randomization by using a computer-number generator, using permuted blocks with variable block sizes from 2 to 6, with blinding to allocation. Block sizes will be selected in random order, with 50 blocks of size 6, 50 blocks of size 4, and 50 blocks of size 2. The Data Management Unit team will transmit participant allocation to the study physician on Day 0. The study staff will not be blinded to participant randomization group. The participant is considered enrolled once randomization occurs.

**9.3.4 Follow-Up Visits**

Study participants will receive all HIV and TB-related care through the study for the first 48 weeks after randomization. The schedule of visits for each group are listed in Appendix 1.

###

### 9.3.5 Reasons for Withdrawal

Participants will be withdrawn from this study for only three reasons. First, participants may withdraw voluntarily from the study at any time if they so desire. Second, the research physician may deem a patient unfit to continue in the study. The major reason for this would be incarceration in prison. The study intervention occurs within the first month of enrollment, and participants will then receive GHESKIO standard of care during the 48-week follow-up period. Poor compliance with study procedures will not be a reason for study exclusion, because retention in care is the primary outcome of the study. If a patient becomes pregnant during the follow-up period, they will receive the same care as that provided to non-study participants at GHESKIO, and will remain in the study unless they request to withdraw. Third, a participant may be withdrawn from the study at the discretion of the protocol chairs, IRBs, data safety monitoring board, or funding source, to ensure that research participants are protected.

###

### 9.3.6 Handling of Withdrawals

The reason for participant withdrawal will be fully documented. Participants will receive standard HIV and TB care free of charge in the GHESKIO clinic.

**9.3.7 Final Study Visit**

The final study visit will occur 48 weeks after HIV testing (Day 0). Participants will be counted as retained in care if they attend the 48-week visit and have blood drawn for HIV-1 RNA testing. As visits are scheduled every 12 weeks at this point in care, a 12-week window period will be permitted for attendance at the final study visit. After completion of the final study visit, participants will be transferred to the routine ART follow-up clinic at GHESKIO for further HIV care.

**10. ASSESSMENT OF SAFETY**

**10.1 Safety Assessment Overview**

Participants in this study will receive standard diagnostic tests, except for the addition of the Xpert Ultra test, which will be commercially available at the time the study begins (and is anticipated to replace the standard Xpert test at GHESKIO and other clinics around the world within the next year). They will also receive standard medication, including ART and TB medications. The difference between standard care and same-day treatment is the timing of receipt of results of diagnostic tests, schedule of visits during the first few weeks of the study, and initiation of either ART or TB medication. It is possible that a diagnosis of TB or another OI could be missed prior to ART initiation in the same-day treatment group, increasing the probability of unmasking IRIS; however it is also possible that this will occur in the standard group. Participants in both groups will receive TB testing that is more intensive than usual care, with both spot and early morning specimens for Xpert Ultra and liquid culture, as well as chest x-ray. If Xpert Ultra is negative, but clinical presentation and chest x-ray are highly suspicious for TB, participants in both groups will receive empiric TB treatment. If liquid culture is positive, participants will be contacted immediately for initiation of TB treatment, if they are not already receiving it.

**10.2 Adverse Event Procedures and Reporting Requirements**

According to the DAIDS Table for Grading the Severity of Adult and Pediatric Adverse Events, Version 2.0 (2014), grading guidelines, an adverse event is “any unfavorable and unintended sign (including an abnormal laboratory finding), symptom, or disease temporally associated with the use of a medical treatment or procedure regardless of whether it is considered related to the medical treatment or procedure.” Adverse events are given a grade from 1 – 5 based on severity of reaction.

Grade 1: This is a mild event that results in mild to no interference in the daily life of the participant. It does not require treatment.

Grade 2: This is a moderate event that results in greater than minimal interference in the daily life of the participant.

Grade 3: This is a severe event resulting in significant inability to perform daily functions.

Grade 4: Grade 4 events are severe, life-threatening events requiring hospitalization.

Grade 5: Indicates death.

We will use the DAIDS Table to grade adverse events, and we will follow these procedures for collecting, recording, evaluating severity, determining expectedness, and assessing the relationship to the study intervention.

1. The adverse event will be documented in the medical record, and the NIH Division of AIDS Expedited Adverse Event Form will be competed for all new DAIDS grade 3 (severe), grade 4 (life-threatening) events, grade 5 (deaths), and for all hospitalizations. This form includes information on severity, expectedness, and relationship to study intervention. Patients with adverse events from ART will be managed according to standard GHESKIO care.
2. The PI, the GHESKIO site PI, and the GHESKIO Director will be notified if any new DAIDS stage 3 or 4 event, death, hospitalization, or complaint regarding loss of confidentiality.
3. For possible medical adverse events, participants will be evaluated within 24 hours (if non-life threatening) or within 2 hours (if life-threatening) of diagnosis of a possible adverse event. The study team will assist patients in receiving immediate medical attention.
4. If any of the following unanticipated problems and adverse events occur, the PI will submit a report within 5 working days (7 calendar days), and submit to IRBs at all participating institutions, a representative of the funding source, and the data safety monitoring board:
   1. Internal adverse events that are unexpected, and related or possibility related to the research, and that indicate there are new or increased risks to participants;
   2. External adverse events that are serious, unexpected, and related or possibly related to the research and that indicate there are new or increased risks to participants that require some action (modification of the protocol, consent process, or informing subjects);
   3. Deviation from the approved research protocol or plan without IRB approval in order to eliminate apparent immediate hazard to subjects or harm to others;
   4. Deviation from the approved research protocol or plan that placed participants at an increased risk of harm regardless of whether actual harm occurred;
   5. Breach of confidentiality;
   6. Any other finding that indicates that there are new or increased risks to participants or that they are less likely to receive any direct benefit from the research.

**11. CLINICAL MANAGEMENT**

Study participants will receive standard TB and ART medication, so adverse events will be those that are shared in standard care. For example, a patient may have an adverse event to ART, or they may develop unmasking IRIS (if a patient presents with subclinical disease or has extrapulmonary TB, which is difficult to diagnose), or paradoxical IRIS. The GHESKIO study team includes highly experienced clinicians, who will manage any adverse events that develop according to standard GHESKIO care, and in the best judgment of the study physicians. Unanticipated and anticipated toxicities will be graded according to the DAIDS Table for Grading the Severity of Adult and Pediatric Adverse Events, Version 2.0, 2014.

Consensus case definitions will be used for paradoxical TB-associated IRIS, ART-associated TB, and unmasking TB-associated IRIS, as proposed by Meintjes et al (Panels 1 and 2 below). For patients without TB, the proposed diagnostic criteria of Haddow et al will be used, with a minor adaptation (unmasking IRIS criteria includes development within 3 months after ART initiation and a marked inflammatory response) [Haddow LJ et a. “Defining Immune Reconstitution Inflammatory Syndrome: Evaluation of an Expert Opinion versus 2 Case Definitions in a South African Cohort”, Clinical Infectious Diseases, 2009; 49:1424-32 (Table 4)].

**Panel 1** ^66^

**Case definition for paradoxical tuberculosis-associated IRIS**

There are three components to this case definition:

(A) Antecedent requirements

Both of the two following requirements must be met:

- Diagnosis of tuberculosis: the tuberculosis diagnosis was made before starting ART and this should fulfill WHO criteria for diagnosis of smear-positive pulmonary tuberculosis, smear-negative pulmonary tuberculosis, or extrapulmonary tuberculosis
- Initial response to tuberculosis treatment: the patient’s condition should have stabilized or improved on appropriate tuberculosis treatment before ART initiation—eg. cessation of night sweats, fevers, cough, weight loss. (Note: this does not apply to patients starting ART within 2 weeks of starting tuberculosis treatment since insufficient time may have elapsed for a clinical response to be reported).

(B) Clinical criteria

The onset of tuberculosis-associated IRIS manifestations should be within 3 months of ART initiation, reinitiation, or regimen change because of treatment failure.

Of the following, at least one major criterion or two minor clinical criteria are required:

*Major criteria*

- New or enlarging lymph nodes, cold abscesses, or other focal tissue involvement—eg, tuberculous arthritis
- New or worsening radiological features of tuberculosis (found by chest radiography, abdominal ultrasonography, CT, or MRI)
- New or worsening CNS tuberculosis (meningitis or focal neurological deficit—eg, caused by tuberculoma)
- New or worsening serositis (pleural effusion, ascites, or pericardial effusion)

*Minor criteria*

- New or worsening constitutional symptoms such as fever, night sweats, or weight loss
- New or worsening respiratory symptoms such as cough, dyspnea, or stridor
- New or worsening abdominal pain accompanied by peritonitis, hepatomegaly, splenomegaly, or abdominal adenopathy

(C) Alternative explanations for clinical deterioration must be excluded if possible*

- Failure of tuberculosis treatment because of tuberculosis drug resistance
- Poor adherence to tuberculosis treatment
- Another opportunistic infection or neoplasm (it is particularly important to exclude an alternative diagnosis in patients with smear-negative pulmonary tuberculosis and extrapulmonary tuberculosis where the initial tuberculosis diagnosis has not been microbiologically confirmed)
- Drug toxicity or reaction

ART=antiretroviral therapy. IRIS=immune reconstitution inflammatory syndrome. *It might be difficult or impossible in resource-poor settings to confirm tuberculosis drug resistance and to exclude certain other infections or neoplasia. Cases where alternative diagnoses cannot be fully excluded because of limited diagnostic capacity should be regarded as “probable paradoxical tuberculosis-associated IRIS”. In these probable cases, should resolution of clinical or radiological findings of the suspected IRIS episode occur without a change in tuberculosis treatment or ART having been made, they could then be reclassified as ”paradoxical tuberculosis-associated IRIS” cases.

**Panel 2** ^67^

**Case definition for ART-associated tuberculosis and provisional case definition for unmasking tuberculosis-associated IRIS**

ART-associated tuberculosis

We propose that ART-associated tuberculosis (all cases of tuberculosis that are diagnosed during ART) should be defined as follows:

- Patient is not receiving treatment for tuberculosis when ART is initiated
- Active tuberculosis is diagnosed after initiation of ART
- The diagnosis of tuberculosis should fulfill WHO criteria for smear-positive

pulmonary tuberculosis, smear-negative pulmonary tuberculosis, or extrapulmonary tuberculosis

Unmasking tuberculosis-associated IRIS (provisional)

We propose that the following could suggest a diagnosis of unmasking tuberculosis-associated IRIS:

- Patient is not receiving treatment for tuberculosis when ART is initiated and then presents with active tuberculosis within 3 months of starting ART

AND one of the following criteria must be met:

- Heightened intensity of clinical manifestations, particularly if there is evidence of a marked inflammatory component to the presentation. Examples include tuberculosis lymphadenitis or tuberculosis abscesses with prominent acute inflammatory features, presentation with pulmonary tuberculosis that is complicated by respiratory failure due to adult respiratory distress syndrome, and those who present with a marked systemic inflammatory syndrome related to tuberculosis.
- Once established on tuberculosis treatment, a clinical course that is complicated by a paradoxical reaction

ART=antiretroviral therapy. IRIS=immune reconstitution inflammatory syndrome. Researchers in the field are encouraged not to regard all patients with ART-associated tuberculosis as having tuberculosis-associated IRIS, but only those that fit this provisional unmasking tuberculosis-associated-IRIS case definition. We suggest that the clinical manifestations of all patients developing ART-associated tuberculosis should be well characterized and reported in studies, which will assist with refinement of this case definition in the future. Studies of the immunological processes underlying the presentation of these cases are also likely to assist with refining this case definition.

**12. STATISTICAL ANALYSIS PLAN**

## Overview and General Design Issues

This is an open-label, randomized study comparing standard care with same-day treatment (ART or TB medication) for symptomatic patients who are newly diagnosed with HIV. A total sample size of 600 participants (300 per arm) will be randomized with equal probability between the two arms. Accrual is anticipated to take 52 weeks (12 months). With a planned follow-up of 48 weeks during the intervention phase for each participant, the accrual and follow-up period is anticipated to be 100 weeks.

## Study Endpoints

**12.2.1 Primary Endpoint**

The primary endpoint is:

- The proportion of participants who are alive and in care with a plasma HIV-1 RNA level <200 copies/ml at 48 weeks after enrollment.

**12.2.2 Secondary Endpoints**

**Secondary efficacy endpoints of this study include:**

1. Mortality at 48 weeks after enrollment, and time to death. Time to death will be measured from the date of randomization to the date of death (whatever the cause).
2. Proportion of participants with HIV-1 RNA level <50 copies/ml and <1000 copies at 48 weeks after enrollment.
3. Proportion of participants with 48-week adherence to ART ≥90%, as measured by medication possession ratio (pharmacy refill records).
4. To document the proportion of participants in each group at the 2-week, 12-week, 24-week, and 48-week visit who report that the last missed dose of medication was at least 2 weeks ago, and to correlate with 24 and 48-week viral load results.
5. Proportion of participants who meet WHO criteria for ART failure, and proportion of patients who start second-line ART.
6. Sensitivity and specificity of spot and early morning Xpert Ultra tests and chest x-ray, as single and as combined tests (in a real-world setting), compared to liquid culture as gold standard. Predictive values and likelihood ratios (positive and negative) will be computed as well.
7. Cost and cost-effectiveness of standard and same-day treatment from the health center perspective.
8. Median time spent in clinic during the first day and month of the study.
9. Mean score on the Connectedness to Treatment Setting Scale, the State Hope Scale, and the COPE survey at 2 weeks, 12 weeks, 24 weeks, and 48 weeks, and mean score on the HRSA Patient Satisfaction Survey at 12, 24, and 48 weeks after enrollment in each group.
10. Barriers and facilitators to treatment initiation, clinic attendance and adherence, assessed through focus groups and in-depth interviews with patients in the standard care and same-day treatment groups.
11. To measure the baseline prevalence of diarrhea (Day 0), and the incidence of diarrhea during the 48-week study period in both groups.
12. Among participants in both groups who report diarrhea, to administer a GHESKIO questionnaire to determine the frequency of diarrhea and the impact on work and other activities.

**Secondary Safety Endpoints:**

1. Incident culture-positive TB after ART initiation. This will be assessed with an automated liquid media culture system (BACTEC MGIT 960, Becton Dickinson).
2. Incidence of paradoxical or unmasking IRIS. The proposed study will use the diagnostic criteria for TB-associated IRIS by Meintjes et al; and for non-TB-related IRIS, by Haddow et al (see Panels 1 and 2 above).^66,67^
3. New Division of AIDS Grade 3 or Grade 4 sign, symptom, or laboratory abnormalities that are at least a one-grade increase from baseline.

## Study Objectives and Hypothesis

## Our primary objective is to evaluate the effectiveness of same-day testing and treatment (TB medication or ART) among symptomatic patients newly diagnosed with HIV. We will test the hypothesis that same-day treatment will result in superior outcomes, compared with standard care, defined as a higher proportion of participants who are alive and in care with a plasma HIV-1 RNA <200 copies/ml at 48 weeks after enrollment. We hypothesize that the proportion of participants with HIV-1 RNA <200 will be higher and that mortality will be lower in the same-day group, because fewer patients will be LTFU prior to treatment initiation. The objective in Aim 3 (secondary aim) is to compare the cost and cost-effectiveness of standard and same-day treatment, where cost is measured by the mean treatment cost and effectiveness is measured by remaining in care with undetectable 48-week viral load.

## Sample Size Considerations

We calculated the sample size for Aim 1 (proportion of participants alive and in care with plasma HIV-1 RNA<200 copies/ml at 48 weeks after enrollment) using the two-sided comparison of proportions. We anticipate a decrease in negative outcomes with same-day treatment, but since this is a new approach, we will look for both increases and decreases in negative outcomes. We based our estimates on current outcomes at GHESKIO with routine care, and the results of the recently completed same-day ART study. Currently at GHESKIO, among patients who present for HIV testing with TB symptoms and who are found to be HIV-infected, 77% of patients remain in care to initiate ART, regardless of whether or not they are diagnosed with TB.

We anticipate a higher ART initiation rate in the standard group, compared with routine clinical care, due to study participation (see Table 1). We anticipate a slight decrease in ART initiation in the same-day group, compared with the original same-day ART study (from 100% to 98%), as ART will be delayed in those diagnosed with TB. We estimate absolute improvements of 5% in both 48-week retention and in viral suppression in both groups, compared with the recently completed same-day ART study, due to expedited follow-up care, which is now provided routinely at GHESKIO. Therefore, for sample size calculations, we estimate that in the standard group, 51% will achieve the primary outcome, compared to 65% in the same-day group. There will be 80% power at a 0.05 significance level to detect this difference with 194 (208) participants per arm using the Chi-square test (Fisher exact test), thus, about 400 participants in the total cohort.  Participants who transfer to another clinic will not be excluded from analyses, so the sample size will not be inflated to account for transfers.

**Table 1: Predicted Patient Outcomes by Group**

|  | **GHESKIO** | **Standard Group** | **Same-Day Group** |
| --- | --- | --- | --- |
| **Completion of TB Testing and ART Initiation**  Proportion of participants who initiate ART (those with TB will receive TB  treatment first, followed by delayed ART, according to WHO guidelines) | 77% | 85% | 98% |
| **48-Week Retention in Care**  Proportion of ART initiators retained in care at 48 weeks  Proportion who started ART and were retained in care for 48 weeks | 72%  55% | 82%  70% | 85%  83% |
| **48-Week Retention in Care with Undetectable Viral Load**  Of those retained, proportion with undetectable viral load (<200 copies/ml)  Proportion of randomized patients who were retained in care with  undetectable 48-week viral load | ----  ---- | 73%  51% | 78%  65% |

For Aim 2 (mortality; secondary aim), we based our estimates on the recently completed same-day ART study. In that study, mortality was 2.3 times higher in the standard group (6.7% vs. 2.9%). For sample size calculations, we anticipate that mortality among patients who remain in care will be similar between the two groups, but slightly higher than in the original same-day ART study (2.5% vs. 2% in the original study). Among patients LTFU in the original same-day ART study, mortality was 20% in the standard group, and 6% in the same-day group; these patients were lost to care, and then with ongoing tracking efforts, were found to have died. For sample size calculations, we will increase mortality among those LTFU by 25% (to 25% in standard and 8% in the same-day group). With a weighted average, this yields a mortality estimate of 10% for the standard group and 4% for the same day group. There will be 80% power at a 0.05 significance level to detect this difference with 283 (315) participants per group using the Chi-square test (Fisher exact test). *Though mortality is a secondary aim, we increased our final target sample size to 300 per group (total sample size of 600).*

## Enrollment and Randomization Procedures

Patients who test HIV-positive and report cough, fever, night sweats, or weight loss on a symptom checklist will be referred to the study staff for screening. After signing the informed consent, they will be evaluated by the study physician and social worker, who will ensure they meet study criteria. Those who meet study criteria and sign the informed consent will be randomized to the standard or same-day treatment groups in a 1:1 ratio using a computer-generated random-number list by the Data Management Team at GHESKIO, as has been done in prior GHESKIO studies.^68^ A data manager in the GHESKIO IT unit who is not involved in the study will conduct randomization by using a computer-number generator, using permuted blocks with variable block sizes from 2 to 6, with blinding to allocation. Block sizes will be selected in random order, with 50 blocks of size 6, 50 blocks of size 4, and 50 blocks of size 2. The Data Management Unit team will transmit participant allocation to the study physician on Day 0. The study staff will not be blinded to participant randomization group. The participant is considered enrolled once randomization occurs.

## Data and Safety Monitoring

A data safety monitoring board (DSMB) has been designated to oversee the safety and effectiveness of the study interventions. This committee will be chaired by Dr. Carlos del Rio, Professor and Chair of the Hubert Department of Global Health at the Rollins School of Public Health and Professor of Medicine in the Division of Infectious Diseases at the Emory University School of Medicine. He is also Co-director for the Clinical Science and International Research Core of the Emory Center for AIDS Research, and is highly experienced in conducting HIV research in resource-poor settings. Dr. Gabriel Timothe, Director General of Haiti’s Ministry of Health, has been on several DSMBs for studies conducted at GHESKIO, and is very experienced in the management of HIV in resource-poor settings. The DSMB will also include Dr. Warren Johnson, B.H. Kean Professor of Tropical Medicine at Weill Cornell Medical College. Dr. Johnson’s career has centered on research and training in resource-poor countries, with a particular focus on tropical diseases and HIV/AIDS. In addition, the DSMB will include Dr. Kenneth Mayer, Director of HIV Prevention at Beth Israel Deaconess Hospital, Medical Research Director of the Fenway Institute, and Professor at Harvard T.H. Chan School of Public Health. Dr. Mayer has a very long history of conducting HIV prevention and treatment studies. A design review meeting will be held with the DSMB prior to implementation of the study. Thereafter, the study will be presented for review by the DSMB at each of their semi-annual meetings. The DSMB will review data related to recruitment, randomization, compliance, and subject safety. Accrual, study conduct (including timeliness and completeness of data collection, fulfillment of eligibility criteria, protocol adherence, trial operating procedures, and form completion), adverse events, and deaths will be monitored. The DSMB will identify needs for additional data, if needed, relevant to safety issues, and make recommendations on the continuation of the study, with regard to recruitment, compliance with protocols, and safety issues. The outcome of each DSMB review will be summarized in a letter to the IRBs of all participating institutions.

## Analysis Plan

All analyses will be conducted with an “intention-to-treat” approach (supplemented by “per-protocol” approach as secondary). We will compare the proportion of participants who are alive and in care with HIV-1 RNA level <200 copies/ml (binary outcome) in the standard and same-day treatment groups using the Chi-square test. The primary analysis will be done without adjusting for baseline variables. If key variables (sex, age, income, education, marital status, WHO stage, body mass index, hemoglobin, and CD4 count) at baseline are notably imbalanced, we will conduct adjusted analyses (as secondary or sensitivity analyses) via multivariable-adjusted logistic regression and Cochran–Mantel–Haenszel statistics. The results will be summarized in terms of proportions as well as odds ratios, along with confidence intervals and statistical significance. Since we have one primary outcome, we will test hypothesis without multiple testing adjustment.

We will compare other binary outcomes using the Chi-square test. We will calculate sensitivity and specificity of spot and early morning Xpert Ultra tests and chest x-ray, as single and combined tests, with liquid culture as the gold standard. We will calculate 95% confidence intervals around sensitivity and specificity using the binomial distribution. We will calculate positive predictive value, negative predictive value, likelihood ratio positive and likelihood ratio negative for each single test and combination of tests. We will compare time spent in clinic (and with each type of provider) during first day and month of the study using the Wilcoxon rank sum test.

Distribution of time-to-event variables will be estimated using the Kaplan-Meier method. Time-to-event distributions between the two treatment strategy arms will be compared with a log rank test. Corresponding hazard ratios (with two-sided confidence intervals) will be estimated using Cox proportional hazards regression models.

We will compare costs and evaluate the cost-effectiveness of standard and same-day treatment, where cost is measured by the mean treatment cost and effectiveness is measured by being in care with viral load <200 copies/ml at 48 weeks after enrollment. We will conduct these analyses from the health system perspective, including the utilization and cost of all health services provided to each participant at GHESKIO and by other providers for the study period, as described in the research plan. Each resource used by each participant during the study period will be multiplied by the unit cost of that item and then summed to determine total costs for the study group. To calculate the mean cost of treatment per participant, we will divide the total cost of treatment for each group by the number of participants randomized to that group. Differences between arms in median and mean costs will be compared using the Wilcoxon rank-sum test and non-parametric bootstrap t-test to account for potential skewness in cost data.

Next, we will conduct a cost-effectiveness analysis, calculating the average cost to produce a participant who is in care and responding to ART, as described in the research plan. We will also calculate the incremental cost-effectiveness ratio (ICER). We will calculate the incremental cost as the difference in total costs between each group, and the incremental effect as the difference in the number of participants who are in care with undetectable viral load. ICER provides an estimate of the resources required to retain one additional patient alive with undetectable viral load. The cost-effectiveness (CE) plane will be constructed with bootstrap samples and 95% CI will be computed. CE acceptability curves will also be drawn in order to understand the probability that same-day treatment is cost-effective for different willingness to pay thresholds. Sensitivity analyses based on the different cost settings (e.g., different cost assumptions, important subgroup analyses) will be performed, from which the most and least favorable scenarios among various real-world settings could be investigated and elucidated.

**13. DATA HANDLING AND RECORD KEEPING**

Demographic, clinical, and laboratory data will be entered into the GHESKIO EMR. An IT programmer and data manager will maintain the EMR database, under the supervision of the research team.

**13.1 Data Management Responsibilities**

The majority of data will be collected as part of clinical care, and will be entered into and maintained in the GHESKIO EMR, as for routine clinical care. The Data Management Team will be responsible for this data, as for routine clinical care at GHESKIO. The only test that is not yet used for clinical care at GHESKIO is the Ultra test (though it is anticipated that the Ultra test will replace standard Xpert within the next year at GHESKIO and in TB clinics around the world). Xpert Ultra results will be entered into the EMR. Additional study forms include an ART readiness survey, the COPE survey, the HRSA Patient Satisfaction Survey, the Connectedness to Treatment Setting Scale, the State Hope Scale, a diarrhea questionnaire, and a form to record 3-day adherence. Additional documentation that will be required is completion of the NIH Division of AIDS Expedited Adverse Event Form for all Grade 3, 4, and 5 adverse events (including deaths), and clinical management forms for the determination of TB risk and management of symptoms at presentation, and management of IRIS.

All data will be de-identified before it is sent to any investigator outside of GHESKIO. Once the data is de-identified, it will be sent to Dr. Heejung Bang at University of California, Davis, and Dr. Pierre Cremieux of Analysis Group, for analysis.

**13.2 Essential/Source Documents and Access to Data/Source Documents**

Source documents will be kept in a secure facility in the GHESKIO Clinical Trials Unit. After completion of the trial, source documents will be moved to a secure room in the outpatient clinic at GHESKIO. They will be kept locked. Documentation from the trial will be kept for five years following total completion of the study. Source data will be available to all monitoring bodies including that require access to ensure patient safety.

**13.3** **Quality Control and Quality Assurance**

Quality control will occur at checkpoints during the study, using two or more people to check that correct data is available. Prior to enrollment, the social worker and research nurse will verify completion of the informed consent form. At the point of enrollment, both the research nurse and data manager will verify that all inclusion and exclusion criteria have been met. During the trial, study coordinators will verify all source documents. A study monitor will review records on a quarterly basis for completeness, document discrepancies, resolve any errors found, monitor Good Clinical Practices and Good Laboratory Practices, and conduct re-trainings as needed.

**14. CLINICAL SITE MONITORING**

A study monitor will conduct quarterly visits to review participant records, including consent forms, CRFs, medical records, and laboratory records to ensure protection of study participants, compliance with the EC/IRB approved protocol/amendments, and accuracy and completeness of records.

**15. HUMAN SUBJECTS PROTECTION**

**15.1 Institutional Review Board/Ethics Committee**

This study will adhere with the ethical standards established by the United States, and the IRBs of GHESKIO, Brigham and Women’s Hospital, Weill Cornell Medical College, and Florida International University. No participant will be enrolled in the study until all associated IRBs have approved it. All participants will sign an informed consent form prior to any involvement in the study. The consent form will describe the purpose of the study, all procedures and activities involved in the study and any anticipated risks or benefits related to participation. A copy of the consent form will be offered to the participant. The original signed consent form will be kept in a secure location at GHESKIO, available at all times to the IRB committee and any monitoring board.

**15.2 VULNERABLE PARTICIPANTS**

**15.2.1 Pregnant women and fetuses:**

Pregnancy is an exclusion criteria for this study. The study intervention takes place during the first month of the study, and participants are then provided with standard care. If a participant becomes pregnant while on the study, they will receive standard care, as for any non-study participant.

**15.2.2 Prisoners**

Prisoners will not be included in the study.

**15.2.3 Children**

Children under 18 years of age will not be included in the study.

**15.2.4 Illiterate Participants**

Many of GHESKIO’s patients live in severe poverty, with limited access to education. Rates of illiteracy are high. The informed consent includes a clear description of the purpose of the study that is understandable in local terms and appropriate for patients who are not able to read or write. For those patients who cannot sign their name, a fingerprint will be accepted.

**15.3 Informed Consent Process**

Patients who are newly diagnosed with HIV, and who report cough, fever, night sweats, or weight loss on the symptom checklist that is administered at HIV testing at GHESKIO will be approached to participate in the study. Prior to any screening procedures, each patient will undergo the informed consent process.

Each potential participant will receive counseling from a GHESKIO social worker. This social worker will explain the purpose of the study, the procedures involved, and the risks and benefits of the study. It will also be explained to the patient that they can withdraw from the study at any time and that this would not compromise their ability to receive standard therapy for HIV and TB from GHESKIO free of charge. Once a patient agrees to participate, the patient will be given a verbal assessment of understanding quiz. A score of 90% or higher will be used to assume appropriate understanding of the study. If a patient receives an appropriate score, then he/she will sign the informed consent form, in the presence of the social worker and research nurse.

**15.4 Risks**

One potential risk of study participation is that a patient could be started on ART while they have undiagnosed TB or another opportunistic infection. For this reason, we have implemented an intensive evaluation for TB, which includes Xpert Ultra testing and liquid culture on both spot and early-morning specimens for both groups. Both groups will also receive chest x-rays, and participants with a clinical syndrome and chest x-ray that is highly suspicious for TB will be treated for TB, regardless of sputum testing results. The level of diagnostic testing for TB in the proposed study is more intensive than that provided for standard GHESKIO patients, and in clinical programs in other resource-poor settings. Other potential causes of IRIS include cytomegalovirus (CMV), pneumocystis, hepatitis B and C, Cryptococcus, and Kaposi’s Sarcoma. These diseases are all rare in Haiti, and they are not part of any standard testing protocol. For CMV and Kaposi’s Sarcoma, standard of care would be to initiate ART as quickly as possible, as treatment options in Haiti are otherwise limited. We have excluded patients with symptoms consistent with Stage 4 neurologic disease, due to the risk of IRIS and other complications from TB meningitis or Cryptococcus. Two of the drugs in the standard ART regimen (tenofovir and lamivudine) are effective against hepatitis B. Testing for pneumocystis is not available in Haiti, and it is a rare disease that is treated empirically.

Though participants in both groups will receive ART literacy and adherence counseling, those in the same-day group could feel psychological stress or discomfort from starting ART on the day of HIV testing. It is possible that some patients will have a difficult time learning their HIV status and starting same-day ART. For this reason, potential participants will meet with a social worker and take the ART Readiness Survey prior to being considered for study enrollment, as described in the research plan. Same-day group participants will also have a follow-up visit in Week 2 for additional adherence counseling and medical evaluation by the study physician. In our original same-day ART study, which was conducted in patients with WHO Stage 1 or 2 diseases, we did not observe difficulty with same-day initiation of ART.

**15.5 Social Impact Events**

Individuals enrolled in this study may experience personal problems resulting from the study participation. Such problems are termed social impact events. Although GHESKIO will make every effort to protect participant privacy and confidentiality, it is possible that participants’ involvement in the study could become known to others, and that participants may experience stigmatization or discrimination as a result of being perceived as being HIV-infected. Problems may also occur in circumstances in which study participation is not disclosed, such as impact on employment related to time taken for study visits.

In the event that a participant reports a social impact event, every effort will be made by study staff to provide appropriate assistance, and/or referrals to appropriate resources. Social impact events are documented and reviewed on a scheduled basis by the protocol team leadership with the goal of reducing their incidence and enhancing the ability of the study staff to mitigate them when possible.

Social impact events that are judged to be serious, unexpected, or more severe or frequent than anticipated, will be reported to all associated IRBs, the DSMB, and the funding source.

**15.6 Benefits**

The potential benefit from participating in this study is that the participant may improve his/her health by being less likely to be lost to care during the study period, if he/she responds positively to the same-day treatment intervention. Participants will also benefit from more intensive TB testing that standard care (Xpert Ultra and liquid culture on both spot and early morning specimens), from closer virologic monitoring than is generally available in Haiti, and from closer proximity to the study team. Participants may also gain an increased awareness of HIV; they may also sense an increased level of concern regarding HIV and social support among the study personnel and the HIV providers.

The interventions in the proposed study may benefit not only the study subjects, but also other HIV-positive individuals in Haiti and other resource-poor settings. If the proposed model of same-day HIV testing and treatment for symptomatic patients is shown to improve treatment initiation rates, retention in care and adherence, then it could serve as a model for other HIV programs in Haiti and around the world.

**15.7 Compensation**

Participants will not be compensated for study participation. They will receive a 100 gourdes ($US 1.70) transportation subsidy at each visit.

**15.8 Participant Privacy and Confidentiality**

All participant-related information including case report forms, laboratory specimens, evaluation forms, reports, etc., will be kept strictly confidential. All records will be kept in a secure, double-locked location and only research staff will have access to their records. Participants will be identified only by means of a coded number specific to each participant. All computerized databases will identify participants by numeric codes only, and will be password-protected. Upon request, participant records will be made available to the study sponsor and other regulatory entities.

**15.9 Study Discontinuation**

The study may be discontinued at any time by the EC/IRB, funder, sponsor, or other government entities as part of their duties to ensure that research participants are protected.

**16. ADMINSTRATIVE PROCEDURES**

This protocol will be registered in ClinicalTrials.gov prior to enrollment of the first participant.

**17. REFERENCES**

1. Zachariah R, Tayler-Smith K, Manzi M, et al. Retention and attrition during the preparation phase and after start of antiretroviral treatment in Thyolo, Malawi, and Kibera, Kenya: implications for programmes? *Trans Roy Soc Trop Med Hy*g 2011;105:421-30.

2. Rosen S, Fox MP. Retention in HIV care between testing and treatment in sub-Saharan Africa: a systematic review. *PLOS Med* 2011;8:e1001056.

3. Noel E, Esperance M, McLaughlin M, et al. Attrition from HIV Testing to Antiretroviral Therapy Initiation among Patients Newly Diagnosed with HIV in Haiti. *J Acquir Immune Defic Syndr* 2012.

4. Tayler-Smith K, Zachariah R, Massaquoi M, et al. Unacceptable attrition among WHO stages 1 and 2 patients in a hospital-based setting in rural Malawi: can we retain such patients within the general health system? *Trans Roy Soc Trop Med Hy*g 2010;104:313-9.

5. Mulissa Z, Jerene D, Lindtjorn B. Patients present earlier and survival has improved, but pre-ART attrition is high in a six-year HIV cohort data from Ethiopia. *PLOS ON*E 2010;5:e13268.

6. Alvarez-Uria G, Pakam R, Midde M, Naik PK. Entry, Retention, and Virological Suppression in an HIV Cohort Study in India: Description of the Cascade of Care and Implications for Reducing HIV-Related Mortality in Low- and Middle-Income Countries. *Interdisc perspect Infect Dis*;2013:384805.

7. McGrath N, Glynn JR, Saul J, et al. What happens to ART-eligible patients who do not start ART? Dropout between screening and ART initiation: a cohort study in Karonga, Malawi. *BMC Pub Health* 2010;10:601.

8. Micek MA, Gimbel-Sherr K, Baptista AJ, et al. Loss to follow-up of adults in public HIV care systems in central Mozambique: identifying obstacles to treatment. *J Acquir Immune Defic Syndr* 2009;52:397-405.

9. Aliyu MH, Blevins M, Parrish DD, et al. Risk factors for delayed initiation of combination antiretroviral therapy in rural north central Nigeria. *J Acquir Immune Defic Syndr* 2014;65:e41-9.

10. Ingle SM, May M, Uebel K, et al. Outcomes in patients waiting for antiretroviral treatment in the Free State Province, South Africa: prospective linkage study. *AIDS* 2010;24:2717-25.

11. Bassett IV, Wang B, Chetty S, et al. Loss to care and death before antiretroviral therapy in Durban, South Africa. *J Acquir Immune Defic Syndr* 2009;51:135-9.

12. Bassett IV, Regan S, Chetty S, et al. Who starts antiretroviral therapy in Durban, South Africa?... not everyone who should. *AIDS* 2010;24 Suppl 1:S37-44.

13. Fox MP, Shearer K, Maskew M, Meyer-Rath G, Clouse K, Sanne I. Attrition through Multiple Stages of Pre-Treatment and ART HIV Care in South Africa. *PLOS One* 2014;9:e110252.

14. Amuron B, Namara G, Birungi J, et al. Mortality and loss-to-follow-up during the pre-treatment period in an antiretroviral therapy programme under normal health service conditions in Uganda. *BMC Pub Health* 2009;9:290.

15. Honge BL, Jespersen S, Nordentoft PB, et al. Loss to follow-up occurs at all stages in the diagnostic and follow-up period among HIV-infected patients in Guinea-Bissau: a 7-year retrospective cohort study. *BMJ Open* 2013;3:e003499.

16. Shastri S, Sathyanarayna S, Nagaraja SB, et al. The journey to antiretroviral therapy in Karnataka, India: who was lost on the road? *J Int AIDS So*c 2013;16:18502.

17. Tayler-Smith K, Zachariah R, Manzi M, et al. Demographic characteristics and opportunistic diseases associated with attrition during preparation for antiretroviral therapy in primary health centres in Kibera, Kenya. *Trop Med Int Health* 2011;16:579-84.

18. Larson BA, Brennan A, McNamara L, et al. Early loss to follow up after enrolment in pre-ART care at a large public clinic in Johannesburg, South Africa. *Trop Med Int Health* 2010;15 Suppl 1:43-7.

19. Kranzer K, Zeinecker J, Ginsberg P, et al. Linkage to HIV care and antiretroviral therapy in Cape Town, South Africa. *PLOS ONE* 2010;5:e13801.

20. Mugglin C, Estill J, Wandeler G, et al. Loss to programme between HIV diagnosis and initiation of antiretroviral therapy in sub-Saharan Africa: systematic review and meta-analysis. *Trop Med Int Health* 2012;17:1509-20.

21. Assefa Y, Van Damme W, Mariam DH, Kloos H. Toward universal access to HIV counseling and testing and antiretroviral treatment in Ethiopia: looking beyond HIV testing and ART initiation. *AIDS Patient Care STDs* 2010;24:521-5.

22. Feldacker C, Johnson D, Hosseinipour M, Phiri S, Tweya H. Who starts? Factors associated with starting antiretroviral therapy among eligible patients in two, public HIV clinics in Lilongwe, Malawi. *PLOS ONE* 2012;7:e50871.

23. Alvarez-Uria G, Midde M, Pakam R, Kannan S, Bachu L, Naik PK. Factors Associated with Late Presentation of HIV and Estimation of Antiretroviral Treatment Need according to CD4 Lymphocyte Count in a Resource-Limited Setting: Data from an HIV Cohort Study in India. *Interdisc perspect Infect Dis* 2012;2012:293795.

24. Fox MP, Rosen S. Retention of Adult Patients on Antiretroviral Therapy in Low- and Middle-Income Countries: Systematic Review and Meta-analysis 2008-2013. *J Acquir Immune Defic Syndr* 2015;69:98-108.

25. Rebeiro PF, Cesar C, Shepherd BE, et al. Assessing the HIV Care Continuum in Latin America: progress in clinical retention, cART use and viral suppression. *J Int AIDS Soc* 2016;19:20636.

26. Toure S, Kouadio B, Seyler C, et al. Rapid scaling-up of antiretroviral therapy in 10,000 adults in Cote d'Ivoire: 2-year outcomes and determinants. *AIDS* 2008;22:873-82.

27. Fairall LR, Bachmann MO, Louwagie GM, et al. Effectiveness of antiretroviral treatment in a South African program: a cohort study. *Arch Intern Med* 2008;168:86-93.

28. Fox MP, Rosen S. Patient retention in antiretroviral therapy programs up to three years on treatment in sub-Saharan Africa, 2007-2009: systematic review. *Trop Med Int Health* 2010;15 Suppl 1:1-15.

29. Charurat M, Oyegunle M, Benjamin R, et al. Patient retention and adherence to antiretrovirals in a large antiretroviral therapy program in Nigeria: a longitudinal analysis for risk factors. *PLOS ONE* 2010;5:e10584.

30. Rosen S, Fox MP, Gill CJ. Patient retention in antiretroviral therapy programs in sub-Saharan Africa: a systematic review. *PLOS Med* 2007;4:e298.

31. Mutasa-Apollo T, Shiraishi RW, Takarinda KC, et al. Patient retention, clinical outcomes and attrition-associated factors of HIV-infected patients enrolled in Zimbabwe's National Antiretroviral Therapy Programme, 2007-2010. *PLOS ONE* 2014;9:e86305.

32. Govindasamy D, Ford N, Kranzer K. Risk factors, barriers and facilitators for linkage to antiretroviral therapy care: a systematic review. *AIDS* 2012;26:2059-67.

33. Geng EH, Bangsberg DR, Musinguzi N, et al. Understanding reasons for and outcomes of patients lost to follow-up in antiretroviral therapy programs in Africa through a sampling-based approach. *J Acquir Immune Defic Syndr* 2010;53:405-11.

34. Koenig S, Dorvil N, Severe P, Riviere C, Faustin M, Perodin C, Paul C, Apollon A, Saintil G, Duverger L, Dumont E, Hedt-Gauthier B, Hennessey K, Rivere V, Devieux J, Pape JW. Same-day HIV testing and antiretroviral therapy initiation results in higher rates of treatment initiation and retention in care. 21st International AIDS Conference, Durban, Oral Presentation. Abstract WEAE0202, 2016.

35. Rosen S, Maskew M, Fox MP, et al. Initiating Antiretroviral Therapy for HIV at a Patient's First Clinic Visit: The RapIT Randomized Controlled Trial. PLoS medicine 2016;13:e1002015.

36. The "START" (a Streamlined ART Initiation Strategy) Study (START-ART). Accessed April 1, 2016 at: <https://clinicaltrials.gov/ct2/show/NCT01810289?term=Geng%2C+Elvin&rank=2>.

37. Steingart KR, Schiller I, Horne DJ, Pai M, Boehme CC, Dendukuri N. Xpert(R) MTB/RIF assay for pulmonary tuberculosis and rifampicin resistance in adults. *Cochrane Database Syst Rev* 2014:CD009593.

38. Theron G, Zijenah L, Chanda D, et al. Feasibility, accuracy, and clinical effect of point-of-care Xpert MTB/RIF testing for tuberculosis in primary-care settings in Africa: a multicentre, randomised, controlled trial. *Lancet* 2014;383:424-35.

39. Automated real-time nucleic acid amplification technology for rapid and simultaneous detection of tuberculosis and rifampin resistance: Xpert MTB/RIF assay for the diagnosis of pulmonary and extrapulmonary TB in adults and children. Policy Update, 2013. World Health Organization.

40. Semitala F IC-FaPDYoXMR, Determine TB-LAM, and Liquid Culture. Abstract WEAB0202. International AIDS Society, Durban, South Africa, 2016.

41. Lawn SD, Brooks SV, Kranzer K, et al. Screening for HIV-associated tuberculosis and rifampicin resistance before antiretroviral therapy using the Xpert MTB/RIF assay: a prospective study. *PLOS Med* 2011;8:e1001067.

42. Cavanaugh JS, Modi S, Musau S, et al. Comparative Yield of Different Diagnostic Tests for Tuberculosis among People Living with HIV in Western Kenya. *PLOS ONE* 2016;11:e0152364.

43. Lawn SD, Kerkhoff AD, Vogt M, Ghebrekristos Y, Whitelaw A, Wood R. Characteristics and early outcomes of patients with Xpert MTB/RIF-negative pulmonary tuberculosis diagnosed during screening before antiretroviral therapy. *Clin Infect Dis* 2012;54:1071-9.

44. Lawn SD, Kerkhoff AD, Vogt M, Wood R. HIV-associated tuberculosis: relationship between disease severity and the sensitivity of new sputum-based and urine-based diagnostic assays. *BMC Med* 2013;11:231.

45. Hanrahan CF, Haguma P, Ochom E, et al. Implementation of Xpert MTB/RIF in Uganda: Missed Opportunities to Improve Diagnosis of Tuberculosis. *Open Forum Infect Dis* 2016;3:ofw068.

46. Albert H, Nathavitharana RR, Isaacs C, Pai M, Denkinger CM, Boehme CC. Development, roll-out and impact of Xpert MTB/RIF for tuberculosis: what lessons have we learnt and how can we do better? *Europ Respir J* 2016;48:516-25.

47. Cox HS, Mbhele S, Mohess N, et al. Impact of Xpert MTB/RIF for TB diagnosis in a primary care clinic with high TB and HIV prevalence in South Africa: a pragmatic randomised trial. *PLOS Med* 2014;11:e1001760.

48. Alland D, Rowneki M, Smith L, Ryan J, Chancellor M et al. Xpert MTB/RIF Ultra: A New Near-Patient TB Test with Sensitivity Equal to Culture. Abstract No. 91. Conference on Retroviruses and Opportunistic Infections, Seattle, Washington, 2015.

49. Manabe YC, Breen R, Perti T, Girardi E, Sterling TR. Unmasked tuberculosis and tuberculosis immune reconstitution inflammatory disease: a disease spectrum after initiation of antiretroviral therapy. *J Infect Dis* 2009;199:437-44.

50. Lai RP, Meintjes G, Wilkinson RJ. HIV-1 tuberculosis-associated immune reconstitution inflammatory syndrome. *Semin Immunopatho*l 2016;38:185-98.

51. Mtei L, Matee M, Herfort O, et al. High rates of clinical and subclinical tuberculosis among HIV-infected ambulatory subjects in Tanzania. *Clin Infect Dis* 2005;40:1500-7.

52. Lawn SD, Myer L, Bekker LG, Wood R. Burden of tuberculosis in an antiretroviral treatment programme in sub-Saharan Africa: impact on treatment outcomes and implications for tuberculosis control. *AIDS* 2006;20:1605-12.

53. Bassett IV, Wang B, Chetty S, et al. Intensive tuberculosis screening for HIV-infected patients starting antiretroviral therapy in Durban, South Africa. *Clin Infect Dis* 2010;51:823-9.

54. Henostroza G, Harris JB, Chitambi R, et al. High prevalence of tuberculosis in newly enrolled HIV patients in Zambia: need for enhanced screening approach. *Int J Tuberc Lung Dis* 2016;20:1033-9.

55. Bonnet MM, Pinoges LL, Varaine FF, et al. Tuberculosis after HAART initiation in HIV-positive patients from five countries with a high tuberculosis burden. *AIDS* 2006;20:1275-9.

56. Koenig SP, Riviere C, Leger P, et al. High mortality among patients with AIDS who received a diagnosis of tuberculosis in the first 3 months of antiretroviral therapy. *Clin Infect Dis* 2009;48:829-31.

57. UNAIDS Fast-Track, Ending the AIDS Epidemic by 2030. Accessed June 7, 2016 at: <http://www.unaids.org/en/resources/campaigns/World-AIDS-Day-Report-2014>.

58. Rosen S, Fox MP, Larson BA, et al. Accelerating the Uptake and Timing of Antiretroviral Therapy Initiation in Sub-Saharan Africa: An Operations Research Agenda. *PLOS Med* 2016;13:e1002106.

59. Ware NC, Wyatt MA, Geng EH, et al. Toward an understanding of disengagement from HIV treatment and care in sub-Saharan Africa: a qualitative study. *PLOS Med* 2013;10:e1001369; discussion e.

60. Bernays S, Rhodes T, Barnett T. Hope: a new way to look at the HIV epidemic. *AIDS* 2007;21 Suppl 5:S5-11.

61. Barnett T, Weston M. Wealth, health, HIV and the economics of hope. *AIDS* 2008;22 Suppl 2:S27-34.

62. Masquillier C, Wouters E, Mortelmans D, Booysen Fle R. Families as catalysts for peer adherence support in enhancing hope for people living with HIV/AIDS in South Africa. *J Int AIDS Soc* 2014;17:18802.

63. Getahun H, Kittikraisak W, Heilig CM, et al. Development of a standardized screening rule for tuberculosis in people living with HIV in resource-constrained settings: individual participant data meta-analysis of observational studies. *PLOS Med* 2011;8:e1000391.

64. Consolidated Guidelines on the Use of Antiretroviral Drugs for Treating and Preventing HIV Infection. Recommendations for a Public Health Approach. June 2013. Geneva, Switzerland: World Health Organization, 2013.

65. Liu C, Zhao Z, Fan J, et al. Quantification of circulating Mycobacterium tuberculosis antigen peptides allows rapid diagnosis of active disease and treatment monitoring. *Proc Natl Acad Sci* 2017;114:3969-74.

66. Haddow LJ, Easterbrook PJ, Mosam A, et al. Defining immune reconstitution inflammatory syndrome: evaluation of expert opinion versus 2 case definitions in a South African cohort. *Clin Infect Dis* 2009;49:1424-32.

67. Meintjes G, Lawn SD, Scano F, et al. Tuberculosis-associated immune reconstitution inflammatory syndrome: case definitions for use in resource-limited settings. *Lancet Infect Dis* 2008;8:516-23.

68. Severe P, Juste MA, Ambroise A, et al. Early versus standard antiretroviral therapy for HIV-infected adults in Haiti. *New Engl J Med* 2010;363:257-65.

**APPENDIX 1 – TABLE OF EVENTS**

**Standard Group – Patient Diagnosed with TB at Initial Testing**

|  | Study Weeks | | | | | | | | | | | | | | | |
| --- | --- | --- | --- | --- | --- | --- | --- | --- | --- | --- | --- | --- | --- | --- | --- | --- |
|  | M1 | | | | | | | | M2 | | M3 | M4 | M5 | M6 | M9 | M12 |
| **Evaluation** | Screening (D0) | Entry (D0) | D2 | D7 | D10 | W2 | W3 | W4 | W6 | W8 | W12 | W16 | W20 | W24 | W36 | W48 |
| HIV test | x |  |  |  |  |  |  |  |  |  |  |  |  |  |  |  |
| CD4 count | x |  |  |  |  |  |  |  |  |  |  |  |  |  |  |  |
| Medical history | x |  |  |  |  |  |  |  |  |  |  |  |  |  |  |  |
| Symptom assessment | x |  |  |  |  | x |  | x | x | x | x | x | x | x | x | x |
| Concomitant medications | x |  |  |  |  | x |  | x | x | x | x | x | x | x | x | x |
| Physical evaluation | x |  |  |  |  | x |  | x | x | x | x | x | x | x | x | x |
| Pregnancy test | x |  |  |  |  |  |  |  |  |  |  |  |  |  |  |  |
| ART readiness survey | x |  |  |  |  |  |  |  |  |  |  |  |  |  |  |  |
| Chest x-ray |  | x |  |  |  |  |  |  |  |  |  |  |  |  |  |  |
| CBC |  | x |  |  |  |  |  |  |  |  |  |  |  |  |  |  |
| Creatinine |  | x |  |  |  |  |  |  |  |  |  |  |  |  |  |  |
| Liver function tests (ALT, AST) |  | x |  |  |  |  |  |  |  |  |  |  |  |  |  |  |
| Xpert Ultra testing (spot ) |  | x |  |  |  |  |  |  |  |  |  |  |  |  |  |  |
| Liquid culture (spot ) |  | x |  |  |  |  |  |  |  |  |  |  |  |  |  |  |
| Xpert Ultra testing (early morning) |  |  | x |  |  |  |  |  |  |  |  |  |  |  |  |  |
| Liquid culture (early morning) |  |  | x |  |  |  |  |  |  |  |  |  |  |  |  |  |
| Store blood for future TB testing |  | x |  |  |  |  |  |  |  |  |  |  |  |  |  |  |
| Store urine for future TB testing |  | x |  |  |  |  |  |  |  |  |  |  |  |  |  |  |
| CRP test |  | x |  |  |  |  |  | x |  |  |  |  |  | x |  |  |
| TB Medication* |  | x | x |  |  | x |  | x | x | x | x | x | x | x |  |  |
| ART Medication* |  |  |  |  |  | x |  | x |  | x | x | x | x | x | x | x |
| Adherence Counseling |  |  |  |  |  | x |  |  |  |  |  |  |  |  |  |  |
| Coping, hope, connectedness, and satisfaction surveys |  |  |  |  |  | x |  |  |  |  | x |  |  | x |  | x |
| Diarrhea questionnaire (if patient has diarrhea) |  | x |  |  |  | x |  | x | x | x | x | x | x | x | x | x |
| HIV-1 RNA |  |  |  |  |  |  |  |  |  |  |  |  |  | x |  | x |

* TB medications will be started as early as Day 0; ART will be started 2 weeks after TB treatment. D=Day; W=Week; M=Months

**Standard Group – Tuberculosis Not Diagnosed at Initial Testing**

| **Evaluation** | Study Weeks | | | | | | | | | | | | | | | |
| --- | --- | --- | --- | --- | --- | --- | --- | --- | --- | --- | --- | --- | --- | --- | --- | --- |
|  | M1 | | | | | | | | M2 | | M3 | M4 | M5 | M6 | M9 | M12 |
|  | Screening (D0) | Entry (D0) | D2 | D7 | D10 | W2 | W3 | W4 | W6 | W8 | W12 | W16 | W20 | W24 | W36 | W48 |
| HIV test | x |  |  |  |  |  |  |  |  |  |  |  |  |  |  |  |
| CD4 count | x |  |  |  |  |  |  |  |  |  |  |  |  |  |  |  |
| Medical history | x |  |  |  |  |  |  |  |  |  |  |  |  |  |  |  |
| Symptom assessment | x |  |  | x |  |  | x |  |  | x | x |  |  | x | x | x |
| Concomitant medications | x |  |  | x |  |  | x |  |  | x | x |  |  | x | x | x |
| Physical evaluation | x |  |  | x |  |  | x |  |  | x | X |  |  | x | x | x |
| Pregnancy test | x |  |  |  |  |  |  |  |  |  |  |  |  |  |  |  |
| ART readiness survey | x |  |  |  |  |  |  |  |  |  |  |  |  |  |  |  |
| Chest x-ray |  | x |  |  |  |  |  |  |  |  |  |  |  |  |  |  |
| CBC |  | x |  |  |  |  |  |  |  |  |  |  |  |  |  |  |
| Creatinine |  | x |  |  |  |  |  |  |  |  |  |  |  |  |  |  |
| Liver function tests (ALT, AST) |  | x |  |  |  |  |  |  |  |  |  |  |  |  |  |  |
| Xpert Ultra testing (spot ) |  | x |  |  |  |  |  |  |  |  |  |  |  |  |  |  |
| Liquid culture (spot ) |  | x |  |  |  |  |  |  |  |  |  |  |  |  |  |  |
| Xpert Ultra testing (early morning) |  |  | x |  |  |  |  |  |  |  |  |  |  |  |  |  |
| Liquid culture (early morning) |  |  | x |  |  |  |  |  |  |  |  |  |  |  |  |  |
| Store blood for future TB testing |  | x |  |  |  |  |  |  |  |  |  |  |  |  |  |  |
| Store urine for future TB testing |  | x |  |  |  |  |  |  |  |  |  |  |  |  |  |  |
| CRP test |  | x |  |  |  |  | x |  |  |  |  |  |  |  |  |  |
| TB Medication* |  |  |  |  |  |  |  |  |  |  |  |  |  |  |  |  |
| ART Medication* |  |  |  | x |  |  | x |  |  | x | x |  |  | x | x | x |
| Adherence Counseling |  |  |  | x |  |  |  |  |  |  |  |  |  |  |  |  |
| Coping, hope, connectedness, and satisfaction surveys |  |  |  |  |  |  | x |  |  |  | x |  |  | x |  | x |
| Diarrhea questionnaire (if patient has diarrhea) |  | x |  |  |  |  | x |  |  | x | x |  |  | x | x | x |
| HIV-1 RNA |  |  |  |  |  |  |  |  |  |  |  |  |  | x |  | x |

* For those who are not diagnosed with TB, ART will be started on Day 10. D=Day; W=Week; M=Months

**Same-day Treatment Group – Patient Diagnosed with TB at Initial Testing**

| **Evaluation** | Study Weeks | | | | | | | | | | | | | | | |
| --- | --- | --- | --- | --- | --- | --- | --- | --- | --- | --- | --- | --- | --- | --- | --- | --- |
|  | M1 | | | | | | | | M2 | | M3 | M4 | M5 | M6 | M9 | M12 |
|  | Screening (D0) | Entry (D0) | D2 | D7 | D10 | W2 | W3 | W4 | W6 | W8 | W12 | W16 | W20 | W24 | W36 | W48 |
| HIV test | x |  |  |  |  |  |  |  |  |  |  |  |  |  |  |  |
| CD4 count | x |  |  |  |  |  |  |  |  |  |  |  |  |  |  |  |
| Medical history | x |  |  |  |  |  |  |  |  |  |  |  |  |  |  |  |
| Symptom assessment | x |  |  |  |  | x |  | x | x | x | x | x | x | x | x | x |
| Concomitant medications | x |  |  |  |  | x |  | x | x | x | x | x | x | x | x | x |
| Physical evaluation | x |  |  |  |  | x |  | x | x | x | x | x | x | x | x | x |
| Pregnancy test | x |  |  |  |  |  |  |  |  |  |  |  |  |  |  |  |
| ART readiness survey | x |  |  |  |  |  |  |  |  |  |  |  |  |  |  |  |
| Chest x-ray |  | x |  |  |  |  |  |  |  |  |  |  |  |  |  |  |
| CBC |  | x |  |  |  |  |  |  |  |  |  |  |  |  |  |  |
| Creatine |  | x |  |  |  |  |  |  |  |  |  |  |  |  |  |  |
| Liver function tests (ALT, AST) |  | x |  |  |  |  |  |  |  |  |  |  |  |  |  |  |
| Xpert Ultra testing  (spot ) |  | x |  |  |  |  |  |  |  |  |  |  |  |  |  |  |
| Liquid culture (spot) |  | x |  |  |  |  |  |  |  |  |  |  |  |  |  |  |
| Xpert Ultra testing (early morning) |  |  | x |  |  |  |  |  |  |  |  |  |  |  |  |  |
| Liquid culture (early morning) |  |  | x |  |  |  |  |  |  |  |  |  |  |  |  |  |
| Store blood for future TB testing |  | x |  |  |  |  |  |  |  |  |  |  |  |  |  |  |
| Store urine for future TB testing |  | x |  |  |  |  |  |  |  |  |  |  |  |  |  |  |
| CRP test |  | x |  |  |  |  |  | x |  |  |  |  |  | x |  |  |
| TB Medication* |  | x |  |  |  | x |  | x | x | x | x | x | x | x |  |  |
| ART Medication* |  |  |  |  |  | x |  | x |  | x | x |  |  | x | x | x |
| Adherence Counseling |  | x |  |  |  |  |  |  |  |  |  |  |  |  |  |  |
| Coping, hope, connectedness, and satisfaction surveys |  |  |  |  |  | x |  |  |  |  | x |  |  | x |  | x |
| Diarrhea questionnaire (if patient has diarrhea) |  | x |  |  |  | x |  | x |  | x | x |  |  | x | x | x |
| HIV-1 RNA |  |  |  |  |  |  |  |  |  |  |  |  |  | x |  | x |
|  | * TB meds will be started on Day 0 with visits every 2 weeks for first 8 weeks and then monthly throughout TB treatment; ART will be started 2 weeks after TB medications. For those without TB, ART will be started on Day 0; D=Day; W=Week; M=Months | | | | | | | | | | | | | | | |

**Same-day Treatment Group – Tuberculosis Not Diagnosed at Initial Testing:**

| **Evaluation** | Study Weeks | | | | | | | | | | | | | | | |
| --- | --- | --- | --- | --- | --- | --- | --- | --- | --- | --- | --- | --- | --- | --- | --- | --- |
|  | M1 | | | | | | | | M2 | | M3 | M4 | M5 | M6 | M9 | M12 |
|  | Screening (D0) | Entry (D0) | D2 | D7 | D10 | W2 | W3 | W4 | W6 | W8 | W12 | W16 | W20 | W24 | W36 | W48 |
| HIV test | x |  |  |  |  |  |  |  |  |  |  |  |  |  |  |  |
| CD4 count | x |  |  |  |  |  |  |  |  |  |  |  |  |  |  |  |
| Medical history | x |  |  |  |  |  |  |  |  |  |  |  |  |  |  |  |
| Symptom assessment | x |  |  |  |  | x |  | x |  | x | x |  |  | x | x | x |
| Concomitant medications | x |  |  |  |  | x |  | x |  | x | x |  |  | x | x | x |
| Physical evaluation | x |  |  |  |  | x |  | x |  | x | x |  |  | x | x | x |
| Pregnancy test | x |  |  |  |  |  |  |  |  |  |  |  |  |  |  |  |
| ART readiness survey | x |  |  |  |  |  |  |  |  |  |  |  |  |  |  |  |
| Chest x-ray |  | x |  |  |  |  |  |  |  |  |  |  |  |  |  |  |
| CBC |  | x |  |  |  |  |  |  |  |  |  |  |  |  |  |  |
| Creatine |  | x |  |  |  |  |  |  |  |  |  |  |  |  |  |  |
| Liver function tests (ALT, AST) |  | x |  |  |  |  |  |  |  |  |  |  |  |  |  |  |
| Xpert Ultra testing  (spot ) |  | x |  |  |  |  |  |  |  |  |  |  |  |  |  |  |
| Liquid culture (spot) |  | x |  |  |  |  |  |  |  |  |  |  |  |  |  |  |
| Xpert Ultra testing (early morning) |  |  | x |  |  |  |  |  |  |  |  |  |  |  |  |  |
| Liquid culture (early morning) |  |  | x |  |  |  |  |  |  |  |  |  |  |  |  |  |
| Store blood for future TB testing |  | x |  |  |  |  |  |  |  |  |  |  |  |  |  |  |
| Store urine for future TB testing |  | x |  |  |  |  |  |  |  |  |  |  |  |  |  |  |
| CRP test |  | x |  |  |  | x |  |  |  |  |  |  |  |  |  |  |
| TB Medication* |  |  |  |  |  |  |  |  |  |  |  |  |  |  |  |  |
| ART Medication* |  |  |  |  |  | x |  | x |  | x | x |  |  | x | x | x |
| Adherence Counseling |  | x |  |  |  |  |  |  |  |  |  |  |  |  |  |  |
| Coping, hope, connectedness, and satisfaction surveys |  |  |  |  |  | x |  |  |  |  | x |  |  | x |  | x |
| Diarrhea questionnaire (if patient has diarrhea) |  | x |  |  |  | x |  | x |  | x | x |  |  | x | x | x |
| HIV-1 RNA |  |  |  |  |  |  |  |  |  |  |  |  |  | x |  | x |
|  | * D=Day; W=Week; M=Months | | | | | | | | | | | | | | | |
