# Supplementary material for: Same-day testing with initiation of antiretroviral therapy or tuberculosis treatment versus standard care for persons presenting with tuberculosis symptoms at HIV diagnosis: A randomized open-label trial from Haiti
Source: PLoS Med. 2023 Jun 9;20(6):e1004246. doi: 10.1371/journal.pmed.1004246 (PMC10292694; doi:10.1371/journal.pmed.1004246)
Supplement: S1 List of Protocol Changes — (DOCX) [file pmed.1004246.s002.docx]

**Protocol Changes:**

Protocol Revisions:

- Protocol Version 2, May 13, 2017
  - Added experimental TB tests for blood and urine samples at baseline
  - Added a secondary outcome on prevalence and incidence of diarrhea, with questionnaire to determine frequency of diarrhea and impact on work and other activities
  - Added a secondary outcome of timing of last missed dose of ART
- Protocol Version 3, December 18, 2017:
  - Changed date of ART initiation for the standard group without TB to Day 7, instead of Day 10, to more closely follow standard procedure.
  - Removed the Day 7 visit for the same-day treatment group (new visit schedule Day 0, 3, 14)
  - Clarified that if a patient presents late in day for HIV testing, or is unable to wait for the confirmatory test result, they may be enrolled, as long as the confirmatory test is conducted within one day after first blood draw, and enrollment occurs on the day of HIV diagnosis.
  - Removed the patient satisfaction survey at Week 2 to reduce the duration of the visit
  - Added additional specimens for the evaluation of predictors of TB recurrence (4mL of blood at baseline and 3mL for patients who complete TB treatment)
  - Replaced Dr. Daniel Fitzgerald with Dr. Warren Johnson on the DSMB

Version 3: Letter of Amendment 1

- Added that first-line ART would be either efavirenz, tenofovir, and lamivudine (as in prior version) or dolutegravir, tenofovir, and lamivudine (which became available in Haiti December 2018.

Version 3: Letter of Amendment 2

- Clarified that patients could be enrolled within 24 hours of receiving their HIV diagnosis
- Clarified that the primary outcome is measured at 48 weeks after enrollment, and that the secondary outcomes of death and additional viral load cut-offs (<50 copies/mL and <1000 copies/mL) are measured at 48 weeks after enrollment
